# Genome-wide analysis of DC1 domain proteins in *Ipomoea* species reveals *IbCHR10* as a positive regulator of salt tolerance in sweet potato

**DOI:** 10.3389/fpls.2026.1780326

**Published:** 2026-03-16

**Authors:** Taifeng Du, Zhen Qin, Yuanyuan Zhou, Zhicheng Jiang, Haiyan Zhang, Fuyun Hou, Liming Zhang

**Affiliations:** 1 Crop Research Institute, Shandong Academy of Agricultural Sciences, Jinan, China; 2 National Center of Technology Innovation for Comprehensive Utilization of Saline-Alkali Land, Dongying, China

**Keywords:** DC1 domain, hormone signaling, *IbCHR10*, salt tolerance, sweet potato

## Abstract

**Introduction:**

Sweet potato (*Ipomoea batatas* (L.) Lam.) is an important food, feed, and industrial crop with high tolerance to marginal environments, yet its complex hexaploid genome limits molecular understanding of stress tolerance mechanisms. DC1 domain proteins, characterized by cysteine/histidine-rich (CHR) zinc-binding motifs, have been implicated in diverse regulatory processes in plants, but their evolutionary features and biological functions remain largely unknown in sweet potato.

**Methods:**

We conducted a genome-wide analysis of the CHR gene family in cultivated sweet potato and its two diploid relatives, *Ipomoea trifida* and *Ipomoea triloba*, including phylogenetic, structural, synteny, promoter cis-element, and expression analyses (transcriptome and qRT-PCR). We further performed functional validation of a candidate gene by overexpression in sweet potato and assessed growth and physiological responses under salt stress.

**Results:**

Twelve *CHR* genes were identified in *I. batatas* and *I. triloba*, and eleven in *I. trifida*. These genes showed conserved gene structures, motif compositions, and syntenic relationships, with segmental duplication contributing to family expansion. Promoter analysis revealed abundant cis-acting elements related to hormone signaling and abiotic stress. Expression analyses demonstrated tissue-specific patterns and strong responses to salt, drought, ABA, and JA treatments. *IbCHR10* was rapidly and strongly induced by salt stress, particularly in a salt-tolerant cultivar. Overexpression of *IbCHR10* enhanced salt tolerance, evidenced by improved growth, reduced oxidative damage, increased antioxidant enzyme activity, enhanced osmotic adjustment, and elevated ABA and JA accumulation under salt stress.

**Discussion:**

This study provides a systematic characterization of DC1 domain (CHR) proteins in *Ipomoea* species and identifies IbCHR10 as an important regulator associated with salt stress tolerance, offering valuable genetic resources for developing stress-resilient sweet potato cultivars.

## 1 Introduction

During their growth and development, plants are continuously exposed to various abiotic stresses, among which soil salinization is considered one of the most severe constraints on global agricultural productivity (Singh, 2021). To cope with salt stress, plants have evolved intricate regulatory networks, within which transcriptional and post-transcriptional regulation serve as rapid and central mechanisms of stress adaptation (Kollist et al., 2019). Numerous transcription factors, including bZIP (He et al., 2024), MYB (Fang et al., 2025), ERF (Li et al., 2022), and WRKY (Li et al., 2021), have been widely reported across diverse plant species to regulate salt-responsive gene expression through hormone-mediated signaling networks, particularly those involving abscisic acid (ABA) and jasmonic acid (JA). Recent studies suggest that a class of proteins containing the DC1 domain participates in plant development and stress responses and may be associated with ABA and JA signaling pathways (Li et al., 2010; Amigo et al., 2025).

The C1 domain is a compact, zinc-binding structural module of approximately 50 amino acids, initially identified in protein kinase C (PKC) isoforms, in which it functions as a lipid-binding motif. Owing to its enrichment in cysteine residues, this domain is also known as the cysteine-rich domain (Das and Rahman, 2014). The C1 domain is essential for the membrane translocation and activation of PKC, which in turn regulates diverse cellular processes-including growth, differentiation, metabolism, and apoptosis-via phosphorylation of downstream targets (Katti et al., 2022). In plants, the divergent C1 (DC1) domain represents a cysteine/histidine-rich (CHR) zinc-finger-like module structurally analogous to animal C1 domains (Arias et al., 2022). However, whether plant DC1 domains retain diacylglycerol (DAG)-binding properties remains unclear, suggesting potential functional divergence between plant DC1 proteins and canonical animal C1 domain-containing proteins (Djafi et al., 2013). DC1 domain proteins have emerged as important regulators of plant development and stress adaptation. For instance, the Arabidopsis DC1 proteins Binucleate Pollen and Vacuoleless Gametophytes are essential for stamen and pollen development (Arias et al., 2022; Brownfield, 2022; D’Ippolito et al., 2017; Amigo et al., 2025); in tomato, SlCHP16 interacts with the 14-3–3 protein TFT12 to modulate floral development (Li et al., 2025). Moreover, TaCHP participates in both ABA-dependent and ABA-independent signaling pathways and enhances salt tolerance by promoting the expression of CBF3 and DREB2A (Li et al., 2010). Arabidopsis At5g17960 has been implicated in multi-hormone-mediated stress responses (Bhaskar et al., 2015), while the cotton miRNA-target module miRNVL5-GhCHR influences salt tolerance through coordinated regulation (Gao et al., 2016). In pepper, CaDC1 enhances resistance to biotic stress by increasing salicylic acid accumulation and activating defense-related genes (Hwang et al., 2014). Collectively, these results highlight the broad functional diversification and evolutionary importance of DC1 domain proteins among diverse plant species. Nevertheless, although advances have been achieved in model plants such as Arabidopsis and tomato, a comprehensive genome-level survey and experimental analysis of DC1 domain proteins in sweet potato remain lacking. Understanding their biological roles is therefore essential for elucidating the molecular basis of stress adaptation in this globally important crop.

Sweet potato (*Ipomoea batatas* (L.) Lam.) is a vital food, feed, and industrial crop that plays an indispensable role in global food security and sustainable agriculture. However, its highly complex hexaploid genome (2n = 6× = 90) poses substantial challenges for dissecting the genetic basis of agronomically important traits (Yan et al., 2022), while simultaneously providing an exceptional system for investigating gene family evolution and functional diversification. Abiotic stress tolerance is a major determinant of sweet potato productivity, and elucidating the underlying regulatory networks is therefore essential for advancing molecular breeding strategies aimed at improving stress resilience.

In this study, we conducted a comprehensive genome-wide analysis of the DC1 domain protein gene family in sweet potato. Based on the genome assemblies of the hexaploid cultivated species *I. batatas* (Yang et al., 2017) and its two diploid wild relatives, *I. trifida* and *I. triloba* (Wu et al., 2018), we systematically identified 12 DC1 domain protein genes (*CHR* genes) in *I. batatas (IbCHRs) and I. triloba* (*ItbCHRs*), and 11 *CHR* genes (*ItfCHRs*) in *I. trifida*. We then examined gene structures, conserved motifs, phylogenetic relationships, and interspecific synteny, and analyzed promoter cis-regulatory elements and expression profiles. Among the identified genes, *IbCHR10* showed the most rapid and sustained induction under salt stress, particularly in a salt-tolerant cultivar, and was therefore selected for functional validation through genetic transformation and phenotypic analysis. Collectively, this study provides the first comprehensive characterization of the DC1 domain protein family in sweet potato, elucidates the evolutionary features and transcriptional dynamics of its members, and establishes the functional relevance of IbCHR10 in salt-stress tolerance. These findings offer valuable genetic resources and mechanistic insights that may facilitate the molecular breeding of stress-resilient sweet potato cultivars.

## 2 Materials and methods

### 2.1 Identification of *CHR* genes in *I. batatas*, *I. trifida*, and *I. triloba*

The genome data of the sweet potato cultivar “Taizhong 6” were downloaded from the “Ipomoea Genome Hub” database (https://sweetpotato.com/, accessed February 7, 2025), while the genome sequences of the diploid wild relatives (*I. trifida* and *I. triloba*) were obtained from the “Sweetpotato Genomics Resource” database (https://sweetpotato.uga.edu/, accessed February 7, 2025). Two complementary approaches were used to identify putative *CHR* genes in the *I. batatas* genome. First, the Hidden Markov Model (HMM) profile of the DC1 domain (PF03107) was retrieved from Pfam database (http://www.ebi.ac.uk/interpro/). Using HMMER v3.4, all putative proteins containing the DC1 domain in the *I. batatas* genome were identified with an E-value cutoff of < 1e^−5^. Second, previously characterized CHR protein sequences from *Arabidopsis thaliana* and *Solanum lycopersicum* were used as queries in BLASTP searches against the *I. batatas* genome (E-value < 1e^−5^). Candidate sequences from both approaches were combined (with redundancy removed) and subjected to domain confirmation using NCBI Batch CD-Search (https://www.ncbi.nlm.nih.gov/, accessed February 11, 2025) and SMART (https://smart.embl.de/, accessed February 11, 2025). Sequences lacking the conserved DC1 domain were excluded from subsequent analyses.

For *I. trifida* and *I. triloba*, CHR proteins were obtained by keyword-based retrieval (Cysteine/Histidine-rich C1 domain) from the “Sweetpotato Genomics Resource” database. All retrieved protein sequences were then verified for the presence of the DC1 domain using NCBI Batch CD-Search and SMART as described above, and sequences lacking the DC1 domain were discarded.

The physicochemical properties of the putative CHR proteins were computed using the ExPASy ProtParam (https://www.expasy.org/, accessed February 15, 2025), including sequence length (aa), molecular weight (MW), theoretical isoelectric point (pI), instability index, aliphatic index, and grand average of hydropathicity (GRAVY). Subcellular localization was predicted using WoLF PSORT (https://wolfpsort.hgc.jp, accessed February 17, 2025).

### 2.2 Chromosomal localization and synteny analysis of *CHR* genes

Chromosomal coordinates of *CHR* genes were obtained from the GFF3 annotation files of *I. batatas, I. trifida*, and *I. triloba*. Based on their physical positions on chromosomes, the genes were renamed sequentially as *IbCHR1*-*IbCHR12*, *ItfCHR1*-*ItfCHR11*, and *ItbCHR1*-*ItbCHR12*, respectively. Interspecies synteny among the three genomes was analyzed using the “One Step MCScanX” module in TBtools (v2.362).

### 2.3 Phylogenetic analysis of CHR proteins

Multiple sequence alignment of CHR proteins (12 from *I. batatas*, 11 from *I. trifida*, 12 from *I. triloba*, 12 from *A. thaliana*, and 21 from *S. lycopersicum*) was performed using MEGA11. A phylogenetic tree was then inferred using the maximum-likelihood method based on the aligned amino acid sequences under the Jones-Taylor-Thornton (JTT) substitution model, with pairwise deletion and 1,000 bootstrap replicates to evaluate branch support.

### 2.4 Gene structure and conserved domain analysis of *CHR* genes

The protein sequences of all putative *CHR* genes from *I. batatas*, *I. trifida*, and *I. triloba* were submitted to the Multiple Expectation Maximization for Motif Elicitation (MEME) suite (version 5.5.3, http://meme-suite.org/, accessed April 5, 2025) for conserved motif discovery. Because CHR proteins are relatively short and their conserved region is dominated by the DC1 domain, the maximum number of motifs was set to 3 to capture the major shared sequence features while avoiding over-fragmentation into redundant motifs; all other parameters were kept at default settings. Furthermore, to confirm the presence of the characteristic DC1 domain, all CHR protein sequences were analyzed using the Conserved Domain Database (CDD) (https://www.ncbi.nlm.nih.gov/Structure/cdd/wrpsb.cgi?SEQUENCE, accessed April 7, 2025). The genomic DNA sequences and corresponding coding sequences (CDS) of *CHR* genes were retrieved from genome annotation files. The exon-intron structure for each gene was visualized and illustrated using TBtools. The number and phases of introns were determined by performing a pairwise alignment between the genomic and CDS sequences for each gene.

### 2.5 Promoter cis-acting elements analysis of *CHR* genes

Promoter sequences (2,000 bp upstream of the translation start codon, ATG) of each *IbCHR*, *ItfCHR*, and *ItbCHR* gene were extracted from the corresponding genome assemblies and submitted to PlantCARE (http://bioinformatics.psb.ugent.be/webtools/plantcare/html/; accessed April 10, 2025) to predict cis-acting regulatory elements (Lescot et al., 2002). Identified elements were manually curated and categorized according to PlantCARE annotations, and the number and type of elements were summarized for each gene. The distribution of major elements across CHR promoters was visualized as a heatmap using TBtools.

### 2.6 Expression pattern analysis of *CHR* genes

To investigate the expression profiles of *ItfCHR* and *ItbCHR* genes, RNA-seq data of *I. trifida* and *I. triloba* across different tissues (flower buds, flowers, leaves, stems, and roots), abiotic stresses (cold, heat, drought, and salt) and hormone treatments (ABA, GA3, and IAA) were downloaded from the “Sweetpotato Genomics Resource” database. All RNA-seq datasets included three independent biological replicates per condition as reported in the original study (Wu et al., 2018). In addition, to analyze the effect of salt stress on the expression of *IbCHR* genes, RNA-seq data from the salt-sensitive cultivar ‘NL54’ and the salt-tolerant cultivar ‘JS26’ at different time points (0 h, 0.5 h, 6 h, and 12 h) under NaCl treatment were obtained from NCBI BioProject PRJNA552932 (Qin et al., 2020). According to the original study, each condition included two independent biological replicates, and differential expression was analyzed using DESeq2. In the present study, the RNA-seq results were primarily used to describe expression dynamics and to prioritize candidate genes for subsequent experimental validation.

For the tissue-specific expression analysis of *IbCHR* genes, samples including shoot apices, leaves, stems, fibrous roots, and storage roots were collected from field-grown sweet potato plants (cv. ‘JS26’). For stress and hormone treatments, stem cuttings (15–20 cm) of ‘JS26’ were cultured hydroponically in Hoagland’s nutrient solution under controlled growth conditions (26 °C, 16 h light/8 h dark). Seedlings were subjected to drought stress using 20% (w/v) PEG6000, and leaves were treated with either 100 μM ABA or 100 μM MeJA; control plants were sprayed with distilled water. Samples were collected at 0, 3, 6, 12, 24, and 48 h after treatment. All samples were immediately frozen in liquid nitrogen and stored at −80 °C until RNA extraction.

Samples from different treatments were ground into a fine powder in liquid nitrogen, and total RNA was extracted using an RNA extraction kit (Vazyme, Nanjing, China). The integrity and concentration of the extracted RNA were assessed by agarose gel electrophoresis and a spectrophotometer (Tiangen, Beijing, China). First-strand cDNA was synthesized from 1 μg of total RNA using a reverse transcription kit (Vazyme, Nanjing, China), according to the manufacturer’s protocol, and used as the template for subsequent qRT-PCR analysis. The reactions were performed on a CFX Connect real-time system (Bio-Rad, USA) with ChamQ Universal SYBR qPCR Master Mix (Vazyme, Nanjing, China). Each 20 μL reaction mixture contained 10 μL of 2×SYBR Mix, 0.8 μL of each gene-specific primer (10 μM), 1 μL of cDNA template, and 7.2 μL of RNase-free water. The amplification conditions were as follows: initial denaturation at 95 °C for 30 s, followed by 40 cycles of 95 °C for 10 s and 60 °C for 30 s. The *IbActin* gene was used as an internal reference to normalize expression levels. Relative gene expression was calculated using the 2^-ΔΔCt^ method. All reactions were performed with three independent biological replicates. The primer sequences used for qRT-PCR are listed in **Supplementary Table 2**.

All heatmaps were generated in TBtools using log2 (x + 1) transformation (log scale base = 2.0; logwith = 1.0) to accommodate zero values, and hierarchical clustering was performed based on the transformed expression matrix.

### 2.7 Subcellular localization of IbCHR10

To determine the subcellular localization of IbCHR10, a transient expression assay was performed in *Nicotiana benthamiana* leaves. Subcellular localization prediction using WoLF PSORT suggested a predominant nuclear localization for IbCHR10. To further evaluate potential targeting signals that might affect GFP-fusion design, sequence-based analyses were performed. Signal peptide prediction using SignalP and organellar transit peptide prediction using TargetP did not identify a canonical N-terminal secretory signal peptide or chloroplast/mitochondrial transit peptide. In addition, inspection of the C-terminal sequence did not reveal a canonical peroxisomal targeting signal or a predicted C-terminal transmembrane helix. The full-length CDS of *IbCHR10* was amplified without the stop codon and fused in-frame to the green fluorescent protein (GFP) at the C-terminus in the pCAMBIA1300-GFP vector under the control of the CaMV 35S promoter, generating the 35S::IbCHR10-GFP construct (primer sequences are provided in **Supplementary Table 2**). The empty vector expressing free GFP (35S::GFP) served as a control. Both constructs were transformed into Agrobacterium tumefaciens strain GV3101. Bacterial cells were resuspended in infiltration buffer (1/2 MS containing 100 μM acetosyringone) to an OD_600_ of 0.6-0.8 and incubated in the dark at 28 °C for 3 h prior to infiltration. The abaxial side of leaves from 4-week-old plants was infiltrated using a needleless syringe, and plants were maintained under low-light conditions for 48–72 h post-infiltration. Nuclei were stained with 4’,6-diamidino-2-phenylindole (DAPI) (1 μg/mL) for 10 min, rinsed with distilled water, and GFP/DAPI signals were observed using a laser scanning confocal microscope.

### 2.8 Generation of *IbCHR10* overexpression transgenic sweet potato plants

The full-length CDS of *IbCHR10* was amplified from cDNA of the salt-tolerant cultivar ‘JS26’ using gene-specific primers (**Supplementary Table 2**) and verified by Sanger sequencing. The ‘JS26’ allele was selected because *IbCHR10* showed strong salt-responsive expression in this cultivar in our RNA-seq profiling. For transformation, cultivar ‘JS33’ was used as the recipient due to its stable regeneration performance and suitability for *Agrobacterium rhizogenes*-mediated transformation in our laboratory.

The *IbCHR10* CDS was cloned into the plant overexpression vector pCAMBIA1300-DsRed, in which *IbCHR10* was driven by the CaMV 35S promoter, while DsRed was expressed from an independent 35S promoter as a reporter cassette. The recombinant construct was introduced into *Agrobacterium rhizogenes* strain K599. Bacteria were cultured to an OD_600_ of 1.0-1.2, collected, and resuspended in MS-based infection medium supplemented with acetosyringone. Transformation was performed using an injection-based method with minor modifications to previously reported protocols. Briefly, healthy virus-free stem segments of cultivar ‘JS33’ were selected, with lateral branches removed while retaining 3–4 fully expanded leaves. The *Agrobacterium* suspension (approximately 1 mL per plant) was injected into nodal regions, and injected stems were soaked overnight in the same infection medium. A total of 20 plants were injected. The inoculated segments were then planted in sterile soil and grown for approximately 90 days. Different tissues were harvested, and putative transformants were preliminarily screened based on red fluorescence. Transgenic plants were confirmed by genomic PCR and qRT-PCR.

### 2.9 Salt tolerance evaluation and physiological analysis of *IbCHR10* overexpressing plants

To assess the salt tolerance of *IbCHR10* overexpressing plants, both wild-type (WT) and *IbCHR10*-OE lines were used for phenotypic and physiological analyses under multiple cultivation systems.

### 2.10 Phenotypic evaluation of *IbCHR10*-OE plants under salt stress

*In vitro* culture experiment: Aseptic tissue culture stem segments from WT and OE plants were cultured on MS solid medium supplemented with 150 mM NaCl. Plant growth performance was recorded after 4 weeks of culture. Pot experiment: Uniform stem cuttings of WT and OE plants were grown in a soil mixture of peat and vermiculite (2:1, v/v) under greenhouse conditions. 4-week-old plants were subjected to salt treatment by irrigation with 200 mM NaCl solution, while control plants received distilled water. Growth performance was evaluated 14 days after treatment. Hydroponic experiment: Seedlings approximately 20 cm in length were transferred to Hoagland’s nutrient solution. After 1 week of acclimation under normal conditions, 200 mM NaCl was added to induce salt stress. Samples were collected 7 days after treatment for subsequent analyses.

#### 2.10.1 Measurement of phenotypic and growth parameters

Root length, fresh weight (FW), and dry weight (DW) of WT and OE plants were recorded following salt treatment. Root length was measured from the tip of the fibrous root to the basal junction. For biomass assessment, plants were immediately weighed after harvest to obtain FW, and then oven-dried at a constant temperature until reaching a stable weight to measure DW.

#### 2.10.2 Determination of physiological and biochemical parameters

Fibrous roots samples from treated plants were collected, frozen in liquid nitrogen, and stored at -80 °C for physiological assays. Malondialdehyde (MDA): MDA concentration was determined using the thiobarbituric acid (TBA) method with a commercial assay kit (Comin, Suzhou, China). Proline: Free proline content was quantified using a proline assay kit (Comin, Suzhou, China) following sulfosalicylic acid extraction and the acid-ninhydrin reaction. Hydrogen peroxide (H_2_O_2_): H_2_O_2_ concentration was measured using a titanium sulfate-based colorimetric assay kit (Comin, Suzhou, China). Superoxide dismutase (SOD) activity: SOD activity was determined by monitoring inhibition of the photochemical reduction of nitroblue tetrazolium (NBT) at 560 nm using a SOD assay kit (Comin, Suzhou, China). ABA and JA: Endogenous ABA and JA levels were quantified using enzyme-linked immunosorbent assay (ELISA) kits as previously described (Caruso et al., 1995). The expression levels of key genes involved in ABA and JA biosynthesis were analyzed by qRT-PCR using gene-specific primers (**Supplementary Table 2**). The *IbActin* gene served as an internal reference, and relative expression levels were calculated using the 2^−ΔΔCt^ method.

## 3 Results

### 3.1 Identification of *CHR* genes in sweet potato and its two diploid wild relatives

A total of 12, 11, and 12 *CHR* genes were identified in *I. batatas*, *I. trifida*, and *I. triloba*, respectively, and were designated as *IbCHR1*-*IbCHR12*, *ItfCHR1*-*ItfCHR11*, and *ItbCHR1*-*ItbCHR12* according to their physical positions on the chromosomes. The basic physicochemical characteristics of these CHR proteins were analyzed (**Supplementary Table 1**). The predicted IbCHR proteins ranged from 127 to 401 amino acids, with molecular weights of 14.11-44.29 kDa, and theoretical isoelectric points (pI) of 6.02-11.44. Notably, 8 IbCHR proteins had pI values > 7, indicating that most IbCHR proteins are predicted to be basic proteins. All IbCHR proteins exhibited instability indices > 40, suggesting that they are intrinsically unstable. Their aliphatic indices ranged from 49.52 to 84.85, and GRAVY values ranged from -0.732 to -0.165, indicating that all IbCHR proteins are predicted to be hydrophilic (GRAVY < 0). Subcellular localization prediction suggested that 6 IbCHR proteins (IbCHR1, IbCHR2, IbCHR4, IbCHR8, IbCHR9, and IbCHR10) are localized nuclear, 2 IbCHR proteins (IbCHR5, and IbCHR7) in peroxisomes, 3 IbCHR proteins (IbCHR6, IbCHR11, and IbCHR12) in mitochondria, and 1 IbCHR protein (IbCHR3) in chloroplast.

In *I. trifida*, the predicted ItfCHR proteins contained 196 to 516 amino acids with molecular weights of 22.15-56.88 kDa and pI values of 5.87-9.37. All ItfCHR proteins showed instability indices > 40, suggesting that they are predicted to be unstable. The aliphatic indices ranged from 58.83 to 75.39, and GRAVY values from -0.564 to -0.234, indicating that these proteins are predicted to be hydrophilic. Subcellular localization prediction suggested that 8 ItfCHR proteins (ItfCHR1, ItfCHR3, ItfCHR4, ItfCHR7-ItfCHR11) are nuclear, ItfCHR2 is cytoplasmic, ItfCHR5 is chloroplastic, and ItfCHR6 is mitochondrial.

Similarly, in *I. triloba*, the predicted ItbCHR proteins ranged from 160 to 419 amino acids, with molecular weights between 17.95 and 43.48 kDa, and pI values of 5.69-9.36. All were predicted to be unstable (instability index > 40). The aliphatic indices (50.31-72.20) and GRAVY values (-0.610 to -0.142) confirmed that all are hydrophilic proteins. Localization prediction indicated that seven ItbCHR proteins (ItbCHR1, ItbCHR3, ItbCHR4, ItbCHR8-ItbCHR11) are nuclear, ItbCHR6 and ItbCHR12 chloroplastic, ItbCHR2 and ItbCHR7 cytoplasmic, and ItbCHR5 mitochondrial.

Collectively, these results indicate that CHR proteins in sweet potato and its diploid relatives share broadly similar physicochemical features, being relatively small, hydrophilic proteins with predicted instability, and showing predominant predicted localization to the nucleus, with additional members targeted to mitochondria, chloroplasts, cytoplasm, or peroxisomes.

### 3.2 Chromosomal distribution and collinearity analysis of *CHR* genes

**Figure 1:**
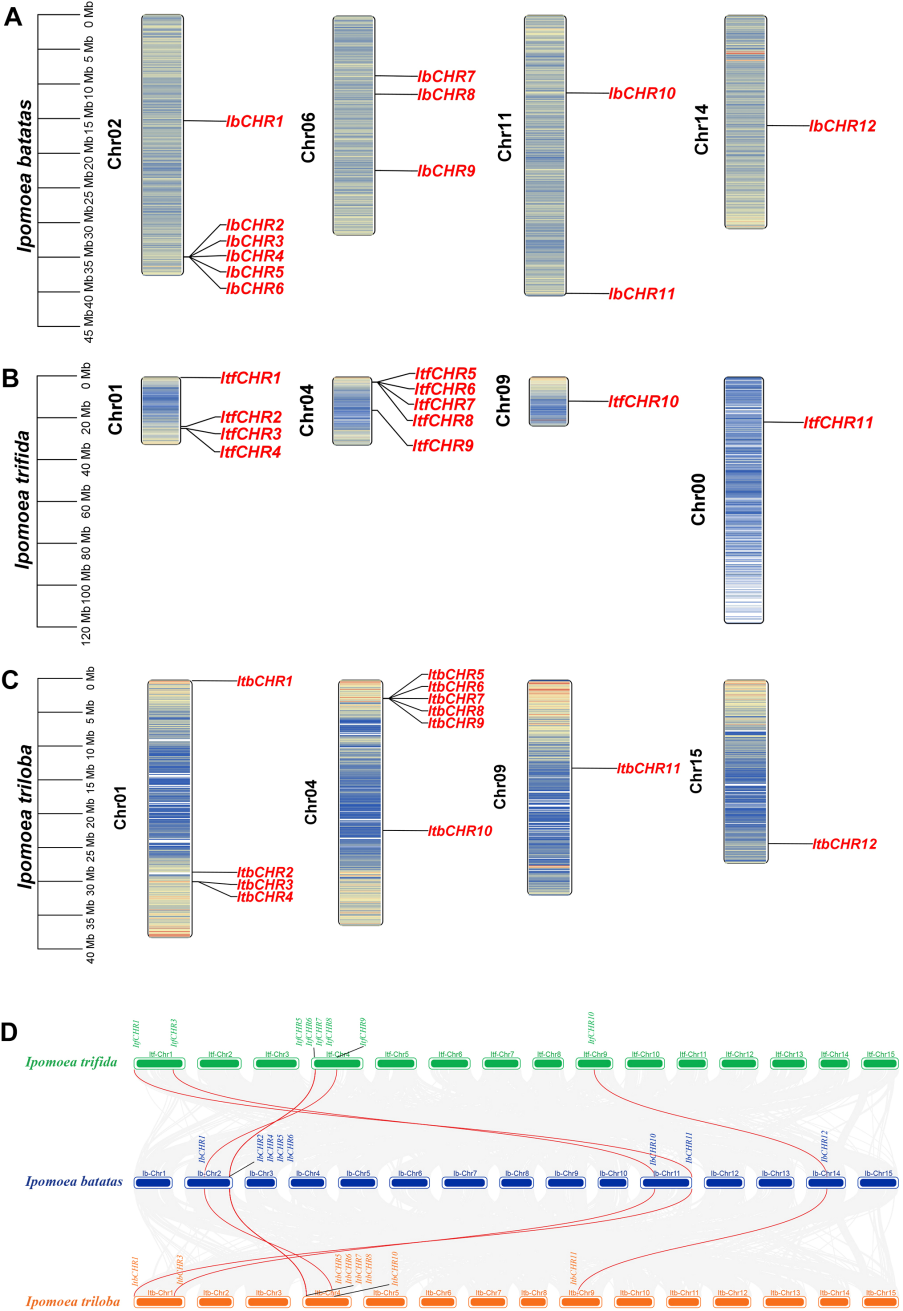
Chromosome localization and synteny of *CHR* genes. **(A)** Chromosomal localization of *IbCHR* genes. **(B)** Chromosomal localization of *ItfCHR* genes. **(C)** Chromosomal localization of *ItbCHR* genes. **(D)** Synteny analysis among *IbCHR*, *ItfCHR*, and *ItbCHR* genes.

Chromosomal mapping revealed that members of the *CHR* gene family in *I. batatas* and *I. triloba* were distributed across 4 chromosomes, while those in *I. trifida* were distributed on 3 chromosomes, except for ItfCHR11, which could not be assigned to any specific chromosome (**Figures 1A–C**). In *I. batatas*, chromosome 2 contained the highest number of *CHR* genes (6 *IbCHR* genes), whereas in *I. trifida* and *I. triloba*, the highest gene density was observed on chromosome 4, with 5 and 6 *CHR* genes, respectively.

Collinearity analysis among the three species revealed that 8 IbCHR genes (*IbCHR1*, *IbCHR3*, *IbCHR5*-*IbCHR10*) exhibited syntenic relationships (**Figure 1D**). Each of these had corresponding orthologs in both *I. trifida* and *I. triloba*, suggesting strong evolutionary conservation during the transition from diploid to hexaploid sweet potato. In contrast, *IbCHR2*, *IbCHR4*, *IbCHR11*, and *IbCHR12* displayed no detectable collinearity with *CHR* genes in the diploid relatives. This absence of synteny implies functional divergence or species-specific expansion associated with polyploidization and adaptive evolution in *I. batatas*.

### 3.3 Phylogenetic analysis of *CHR* genes

**Figure 2:**
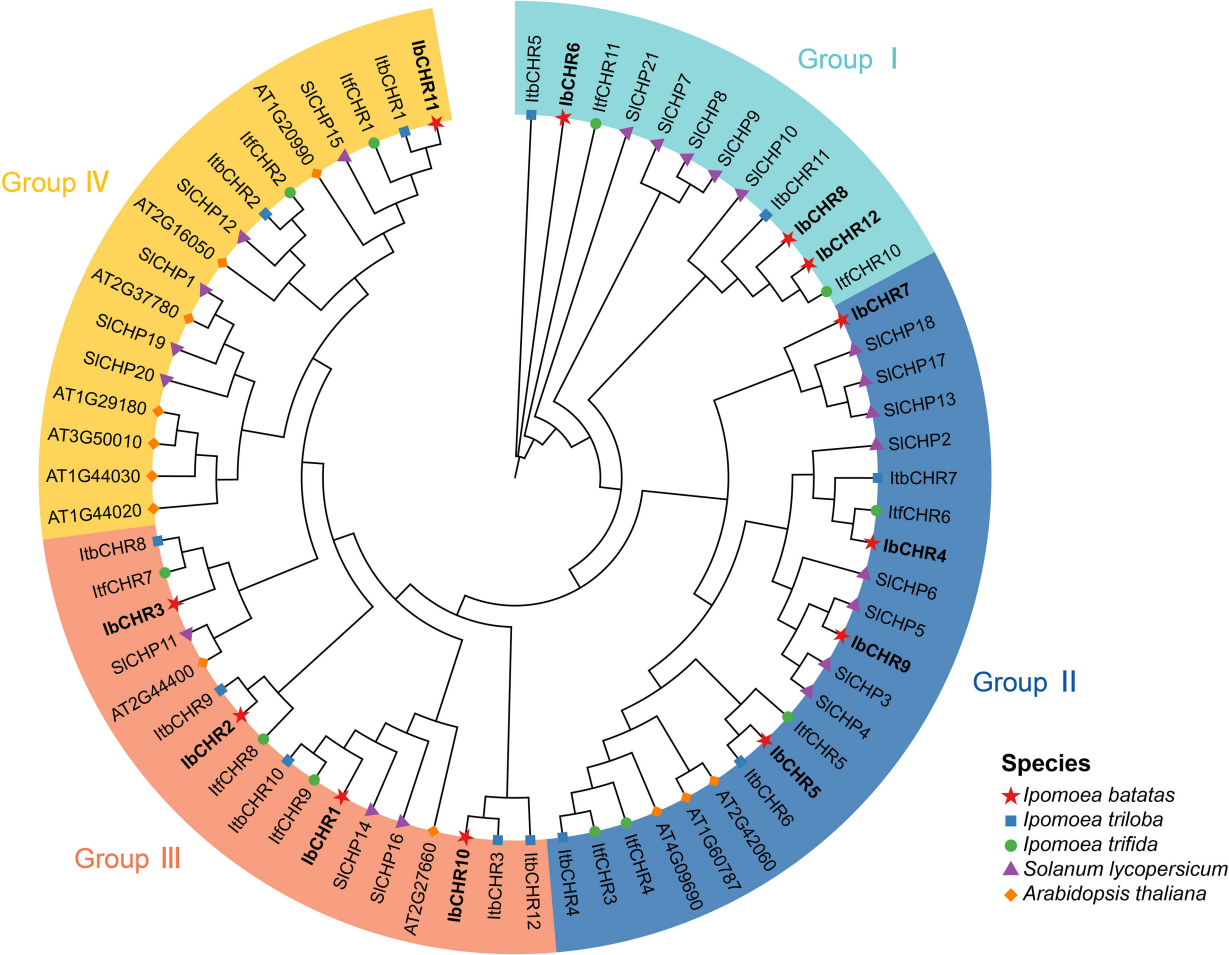
Phylogenetic analysis of CHR proteins. The tree was inferred in MEGA11 using full-length amino acid sequences of 12 IbCHR, 12 ItbCHR, 11 ItfCHR, 12 AtCHR, and 21 SlCHR proteins with the maximum-likelihood method under the JTT substitution model and 1,000 bootstrap replicates. CHR proteins were classified into four major groups (different colors). Marker shapes indicate proteins from different species.

To investigate the evolutionary relationships of CHR proteins, a phylogenetic tree was constructed using 68 CHR protein sequences from *I. batatas* (12), *I. trifida* (11), *I. triloba* (12), *A. thaliana* (12), and *S. lycopersicum* (21). Based on the phylogenetic topology, these genes clustered into four major groups (**Figure 2**). Group I comprised 3 CHR proteins from *I. batatas*, 2 from *I. trifida*, 2 from *I. triloba*, and 5 from *S. lycopersicum*. Group II included 4 IbCHR proteins, 4 ItfCHR proteins, 3 ItbCHR proteins, 8 SlCHR proteins, and 3 AtCHR proteins. Group III contained 4 IbCHR proteins, 3 ItfCHR proteins, 5 ItbCHR proteins, 3 SlCHR proteins, and 2 AtCHR proteins. Finally, Group IV consisted of 1 IbCHR protein, 2 ItfCHR proteins, 2 ItbCHR proteins, 5 SlCHR proteins, and 7 AtCHR proteins. The uneven distribution of CHR proteins across clades suggests lineage-specific expansion and potential functional diversification during plant evolution.

### 3.4 Conserved motif and gene structure analysis of the *CHR* genes

**Figure 3:**
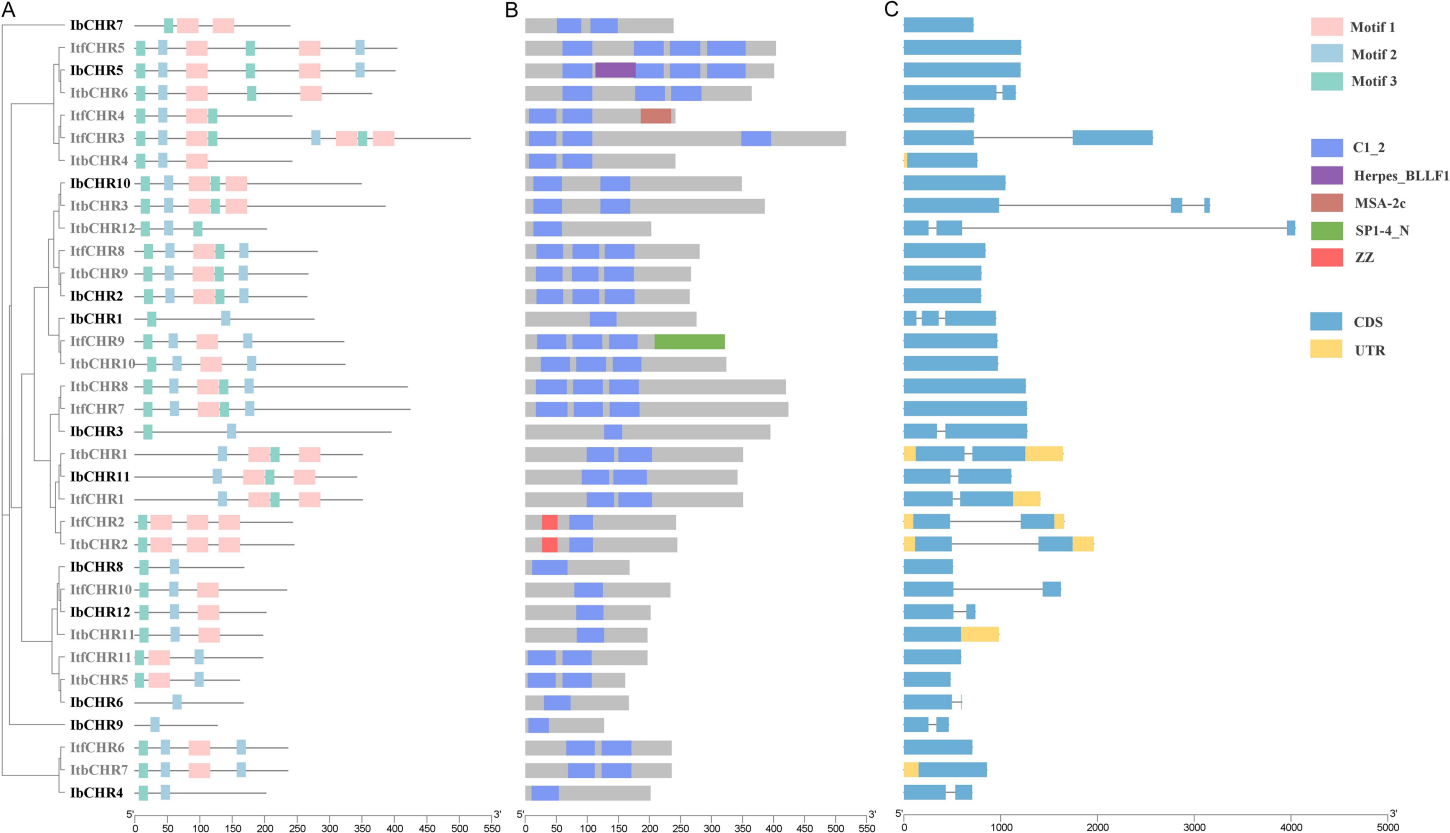
Conserved motifs, domain architecture, and gene structures of *CHR* genes. **(A)** Distribution of conserved motifs (1-3) identified by MEME in CHR proteins. **(B)** DC1 domain architecture of each CHR protein as annotated by CDD (blue boxes). **(C)** Exon-intron structures of *CHR* genes. Blue boxes represent CDS, yellow boxes represent UTR, and black lines represent introns.

The conserved motifs and exon-intron architectures of *CHR* genes were examined to evaluate structural diversity (**Figure 3**). Using MEME, 3 conserved motifs were identified among CHR proteins from *I. batatas*, *I. trifida*, and *I. triloba* (**Supplementary Figure 1**). Motif 1 was absent in ItbCHR12, IbCHR1, IbCHR3, IbCHR4, IbCHR6, IbCHR8, and IbCHR9, whereas Motif 2 was missing in ItfCHR2, ItbCHR2, and IbCHR7; Motif 3 was not detected in IbCHR6 or IbCHR9. CDD analysis confirmed that all CHR family members contain a conserved DC1 domain, supporting their classification as DC1/CHR proteins. Gene structure analysis revealed that most *CHR* genes possessed a simple exon-intron organization, with a predominance of intronless members. The majority of *CHR* genes (19) were intronless, while 13 *CHR* genes contained a single intron. Only a few genes, such as *ItbCHR3*, *ItbCHR12*, and *IbCHR1*, harbored two introns. These findings indicate that although *CHR* genes are evolutionarily conserved at the domain level, they exhibit structural diversification in exon-intron organization, which may contribute to their functional divergence among species.

### 3.5 Cis-acting element analysis of *CHR* gene promoters

**Figure 4:**
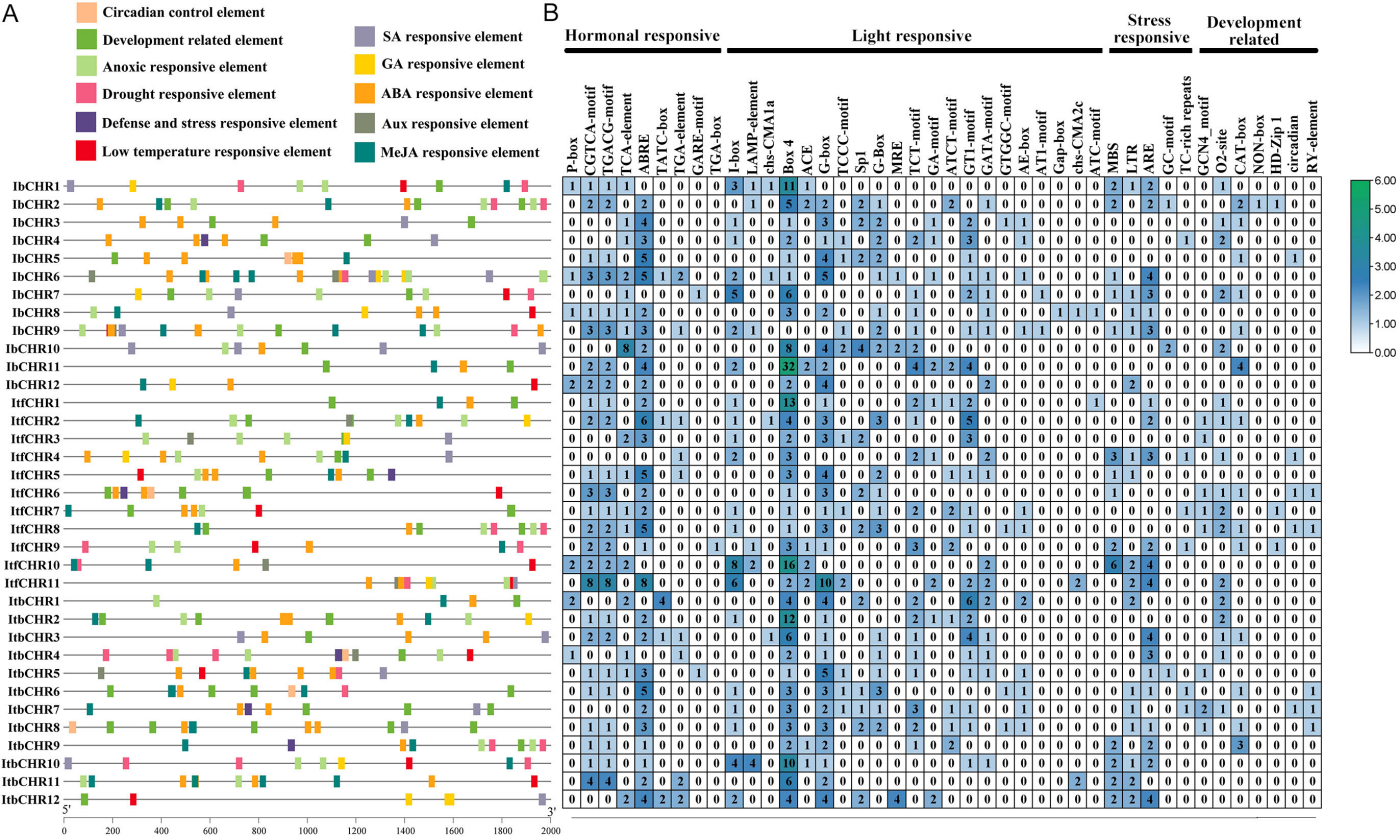
Cis-acting elements analysis of *CHR* promoters. **(A)** Schematic diagram of the distribution of major cis-acting elements in the promoter regions of CHR genes. Different colored boxes represent different categories of regulatory elements, including those involved in hormone response, stress response, and plant development. **(B)** Heatmap showing the occurrence frequency of cis-elements across *CHR* genes promoters; color intensity reflects element counts.

To explore potential transcriptional regulation of *CHR* genes, cis-acting regulatory elements were analyzed within the 2000 bp upstream promoter regions of *IbCHR*, *ItfCHR*, and *ItbCHR* genes using the PlantCARE database. A total of 992 cis-acting elements were identified and classified into 4 categories: light-responsive elements, hormone-responsive elements, stress-responsive elements, and development-related elements (**Figure 4**). Light-responsive elements were found to be widely distributed across the promoters of all *CHR* genes, accounting for a total of 563 elements. Notably, the *IbCHR11* promoter contained the highest number of light-responsive elements (50), suggesting a potential role in photosynthetic regulation. Hormone-responsive elements were also abundant, with MeJA-responsive elements (CGTCA-motif) and ABA-responsive (ABRE) elements being the most frequent, present in the promoters of 49 and 29 genes, respectively. In addition, 109 stress-responsive elements were detected, including ARE (anaerobic induction), TC-rich repeats (defense and stress response), and LTR (low-temperature response). Collectively, these results indicate that *CHR* genes are regulated by complex hormonal and environmental cues, implying that they may participate in hormone-mediated and stress-adaptive regulatory networks that contribute to the environmental resilience of sweet potato and its relatives.

### 3.6 Expression pattern analysis of *CHR* genes

#### 3.6.1 Expression patterns analysis of *CHR* genes in various tissues

**Figure 5:**
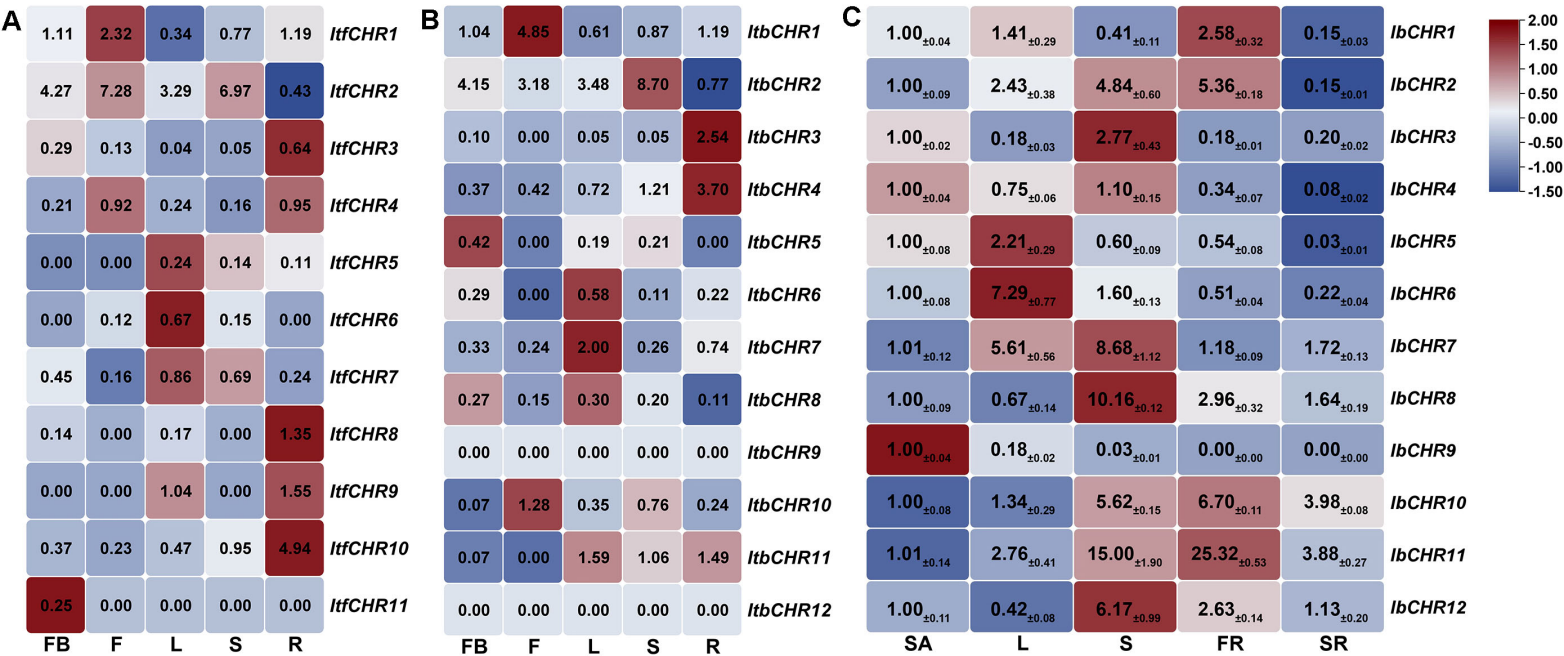
Expression profiles of *CHR* genes in different tissues. **(A)** RNA-seq-based expression patterns of *ItfCHR* genes across flower buds, flowers, leaves, stems, and roots in *I. trifida*. **(B)** RNA-seq-based expression patterns of *ItbCHR* genes across the same tissues in *I. triloba*. For **(A, B)**, expression values are FPKM from the original dataset (Wu et al., 2018) and are shown as the mean of three independent biological replicates after log2(FPKM + 1) transformation for heatmap visualization. **(C)** qRT-PCR analysis of *IbCHR* expression in shoot apices, leaves, stems, fibrous roots, and storage roots of *I. batatas*. Expression levels were normalized to *IbActin* and are presented relative to shoot apices (SA). Values represent mean ± SD of three independent biological replicates (each replicate pooled from three plants) calculated using the 2^-ΔΔCt^ method.

To assess tissue-associated expression patterns of *ItfCHR* and *ItbCHR* genes, we analyzed public RNA-seq datasets from five tissues, including flower buds (FB), flowers (F), leaves (L), stems (S), and roots (R) (**Figures 5A, B**). In these datasets, expression was quantified as fragments per kilobase per million mapped reads (FPKM) in the original study, and the heatmaps display mean FPKM values across three independent biological replicates after log2(FPKM + 1) transformation to accommodate zero values (Wu et al., 2018). In this study, terms such as “higher” or “lower” expression refer to relative differences in mean normalized expression (FPKM) across tissues/treatments as visualized in the log2(FPKM + 1) heatmaps, rather than statistical differential expression.

In *I. trifida*, several genes showed higher relative expression in specific tissues, with *ItfCHR1* and *ItfCHR2* exhibiting comparatively higher expression in flowers, *ItfCHR5–ItfCHR7* in leaves, and *ItfCHR3*, *ItfCHR4*, and *ItfCHR8–ItfCHR10* in roots; *ItfCHR11* showed detectable expression mainly in flower buds (**Figure 5A**). In *I. triloba*, *ItbCHR5* displayed higher expression in flower buds, *ItbCHR1* and *ItbCHR10* in flowers, and *ItbCHR6–ItbCHR8* and *ItbCHR11* in leaves; *ItbCHR2* showed higher expression in stems, whereas *ItbCHR3* and *ItbCHR4* exhibited higher expression in roots (**Figure 5B**). Notably, *ItbCHR9* and *ItbCHR12* showed FPKM values of 0 across the sampled tissues in this dataset, indicating no detectable transcription under these conditions; however, low-level expression below the detection/quantification threshold cannot be excluded.

**Figure 6:**
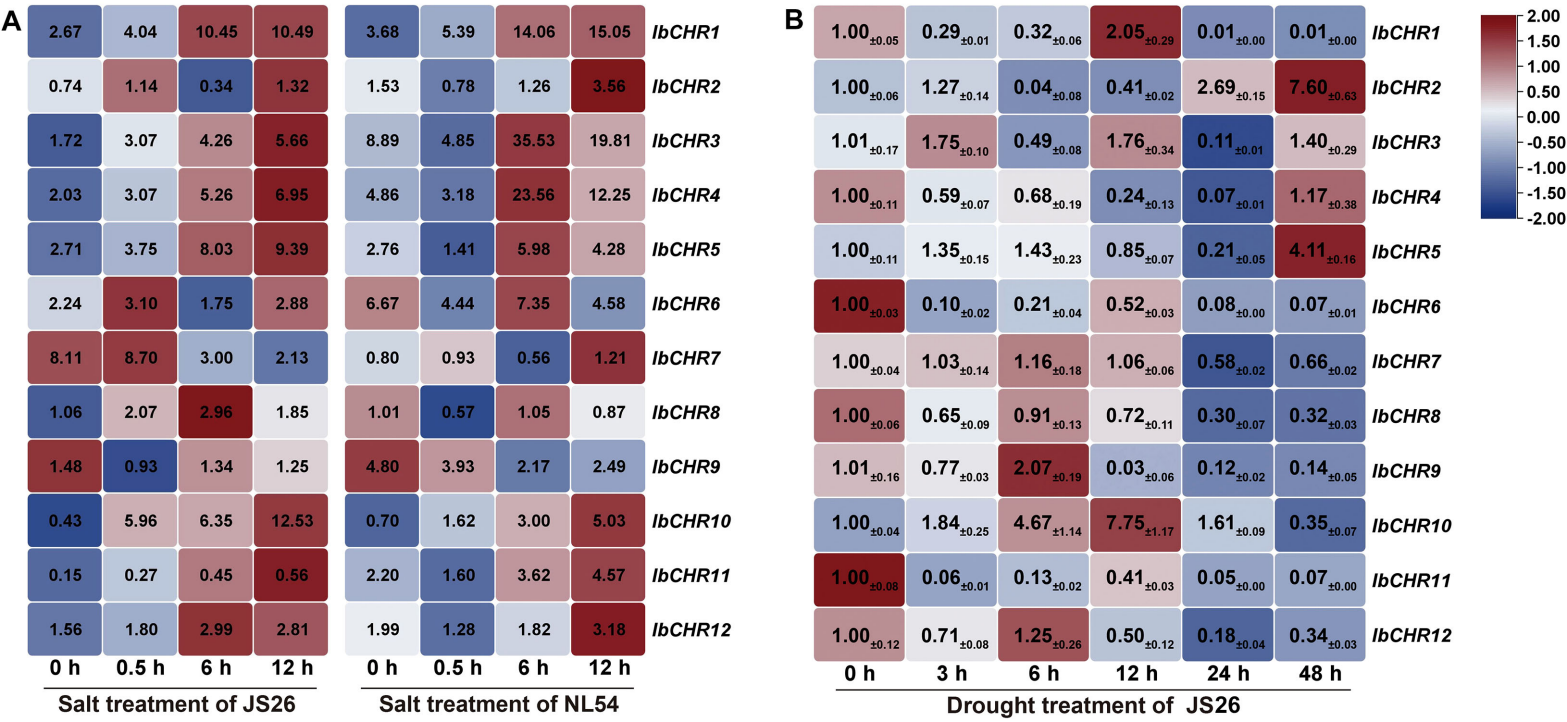
Expression patterns of *IbCHR* genes under salt and drought stresses. **(A)** RNA-seq–based time-course expression profiles of *IbCHR* genes in the salt-tolerant cultivar ‘JS26’ and the salt-sensitive cultivar ‘NL54’ at 0, 0.5, 6, and 12 h after NaCl treatment (Qin et al., 2020). Expression values are FPKM from the original dataset, which included two independent biological replicates per condition. Heatmaps were generated in TBtools using the mean FPKM values across replicates and log2(FPKM + 1) transformation (base = 2.0; logwith = 1.0). **(B)** qRT-PCR–based expression dynamics of *IbCHR* genes in ‘JS26’ under PEG6000 treatment at the indicated time points. Expression levels were normalized to *IbActin* and are presented relative to 0 h. Values represent mean ± SD of three independent biological replicates. Heatmap color intensity indicates relative expression.

For *I. batatas*, tissue expression of *IbCHR* genes was examined by qRT-PCR in shoot apices (SA), leaves (L), stems (S), fibrous roots (FR), and storage roots (SR) (**Figure 5C**). Overall, several *IbCHR* genes exhibited higher relative expression in vegetative organs, with *IbCHR9* showing higher expression in shoot apices, *IbCHR5* and *IbCHR6* in leaves, *IbCHR3*, *IbCHR4*, *IbCHR7*, *IbCHR8*, and *IbCHR12* in stems, and *IbCHR1*, *IbCHR2*, *IbCHR10*, and *IbCHR11* in fibrous roots. In contrast, most *IbCHR* genes showed relatively lower expression in storage roots. Because tissue expression patterns for *I. trifida/I. triloba* were derived from RNA-seq (FPKM-based) datasets whereas those for *I. batatas* were determined by qRT-PCR, comparisons across species should be interpreted qualitatively rather than as direct quantitative contrasts. RNA-seq datasets were used for cross-condition trend inspection, whereas qRT-PCR was performed to validate expression patterns of *IbCHR* genes in our materials under controlled treatments.

#### 3.6.2 Expression patterns under abiotic stresses and hormone treatments

To examine the potential involvement of *ItfCHR* and *ItbCHR* genes in abiotic stress and hormone responses, we analyzed public RNA-seq datasets generated under cold, heat, drought, and salt treatments, as well as ABA, GA3, and IAA applications (**Supplementary Figure 2**). In the original study, expression was quantified as FPKM and each condition included three independent biological replicates (Wu et al., 2018). Heatmaps in **Supplementary Figure 2** were used to visualize relative expression patterns across treatments (log2-transformed as described in Materials and Methods); Values of 0 (FPKM = 0) indicate that transcripts were not detected in the corresponding dataset under the tested conditions.

In *I. trifida*, several *ItfCHR* genes displayed treatment-associated changes relative to their corresponding controls (**Supplementary Figure 2A**). Under cold stress, multiple genes (e.g., *ItfCHR2*, *ItfCHR4*, *ItfCHR6*, and *ItfCHR9*) showed higher transcript abundance compared with the control, whereas *ItfCHR1* and *ItfCHR10* showed lower levels. Heat treatment was associated with increased expression of *ItfCHR6* and *ItfCHR9*, while several genes showed little change or reduced expression. Under drought and salt treatments, *ItfCHR1* and *ItfCHR2* exhibited increased expression relative to the control, whereas *ItfCHR6*, *ItfCHR7*, and *ItfCHR10* tended to decrease; *ItfCHR9* showed an increase under both treatments. For hormone treatments, *ItfCHR1* showed a pronounced increase under ABA, while GA3 and IAA elicited gene-dependent responses, including higher expression of *ItfCHR2* under IAA (**Supplementary Figure 2A**). Notably, *ItfCHR3* showed no detectable expression across the tested conditions in this dataset.

In *I. triloba*, cold stress was associated with elevated expression of several genes, including strong increases in *ItbCHR4*, *ItbCHR6*, and *ItbCHR7*, whereas *ItbCHR1* showed a slight decrease compared with the control (**Supplementary Figure 2B**). Heat treatment was accompanied by higher expression of *ItbCHR4* and *ItbCHR7–ItbCHR9* relative to the control. Under drought and salt stress, *ItbCHR1* and *ItbCHR2* showed increased expression, while some genes exhibited treatment-specific differences (e.g., *ItbCHR4* decreased under salt compared with its control). ABA treatment was associated with higher expression of *ItbCHR1*, *ItbCHR2*, and *ItbCHR10*, whereas GA3 and IAA induced distinct, gene-specific expression patterns (**Supplementary Figure 2B**). *ItbCHR5* and *ItbCHR12* showed little to no detectable expression in most conditions in this dataset.

#### 3.6.3 Expression patterns of *IbCHR* genes under salt and drought stresses

To investigate the expression dynamics of *IbCHR* genes under salt stress, RNA-seq data from the salt-sensitive cultivar ‘NL54’ and the salt-tolerant cultivar ‘JS26’ sampled at 0, 0.5, 6, and 12 h after NaCl treatment were analyzed (**Figure 6A**). In the original dataset, expression was quantified as FPKM with two independent biological replicates per condition (Qin et al., 2020). In the present study, RNA-seq results were used primarily to describe temporal expression trends and to prioritize candidate genes for subsequent experimental validation; for visualization, heatmaps were generated in TBtools using the mean FPKM values across replicates after log2(FPKM + 1) transformation. Several genes, including *IbCHR1*, *IbCHR10*, and *IbCHR11*, showed increased transcript abundance over time following salt treatment in both cultivars, with *IbCHR10* exhibiting a more pronounced increase in ‘JS26’ than in ‘NL54’, consistent with its selection as a salt-responsive candidate associated with tolerance. Notably, *IbCHR7* displayed divergent temporal patterns between the two cultivars, with reduced expression in ‘JS26’ but higher expression in ‘NL54’ at later time points, suggesting genotype-associated regulatory differences.

Under drought stress (PEG6000 treatment), qRT-PCR analysis in ‘JS26’ revealed distinct time-dependent expression patterns (**Figure 6B**). *IbCHR2*, *IbCHR4*, and *IbCHR5* showed an early decrease followed by a gradual increase toward later time points, reaching their highest levels at 48 h. In contrast, *IbCHR7*, *IbCHR9*, and *IbCHR10* increased during early stages and then declined, with the highest levels generally observed around 6–12 h. In addition, *IbCHR8* exhibited a sustained decrease throughout the treatment period. Collectively, these results indicate that *IbCHR* genes exhibit dynamic, time-dependent transcriptional responses to salt and drought-related stimuli in sweet potato.

#### 3.6.4 Expression patterns of *IbCHR* genes under hormone treatments

**Figure 7:**
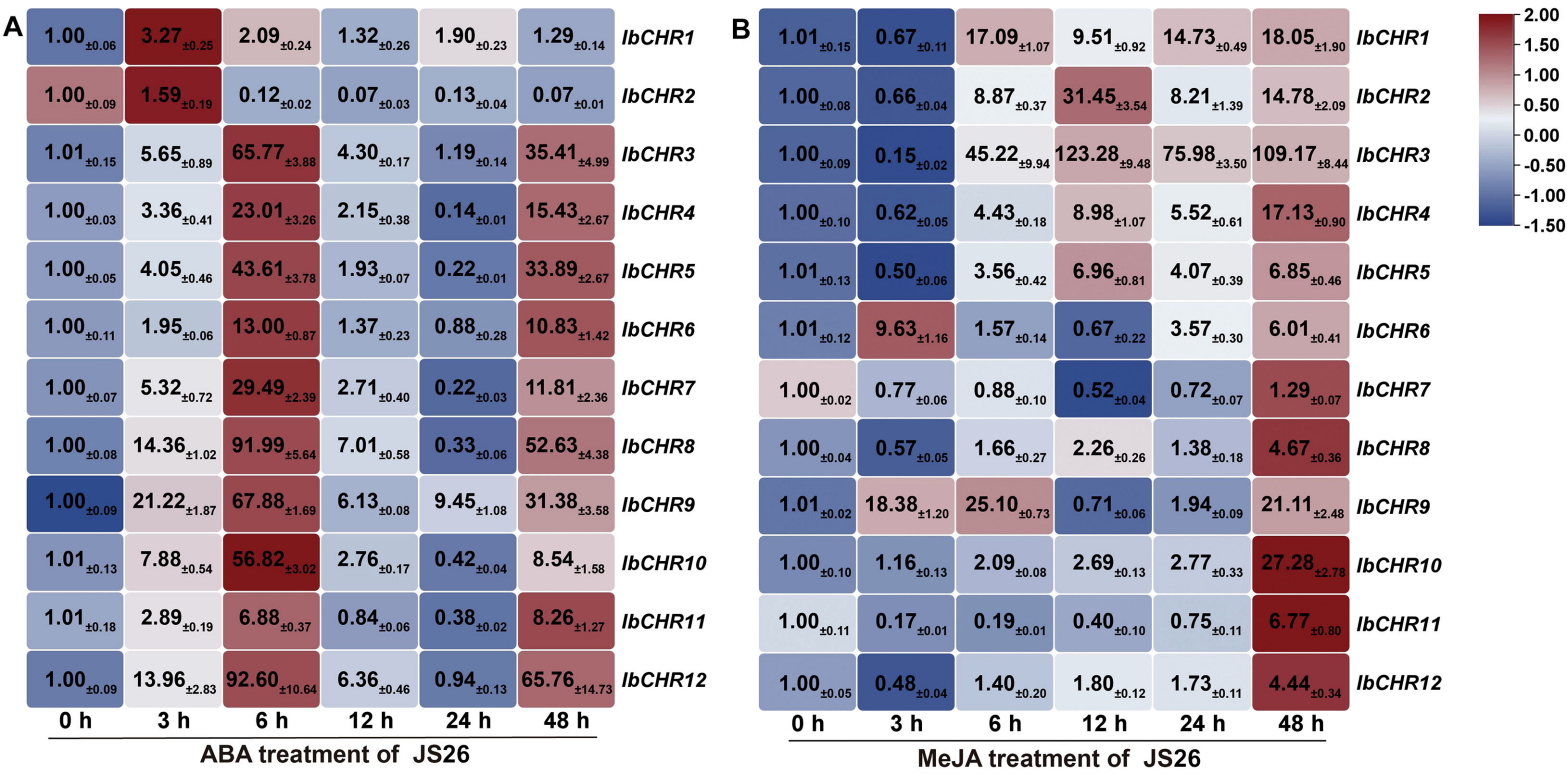
Hormone-responsive expression patterns of *IbCHR* genes. **(A)** qRT-PCR-based time-course expression profiles of *IbCHR* genes following ABA treatment. **(B)** qRT-PCR-based expression dynamics following MeJA treatment. For both panels, transcript levels were normalized to *IbActin* and are presented relative to 0 h. Values represent mean ± SD of three independent biological replicates. Heatmaps were generated in TBtools using the mean relative expression values and log2(x + 1) transformation for visualization.

To further examine hormone responsiveness, the expression of *IbCHR* genes following ABA and MeJA treatments was analyzed by qRT-PCR at 0, 3, 6, 12, 24, and 48 h (**Figure 7**). Under ABA treatment, *IbCHR1* and *IbCHR2* showed an early increase at 3 h and then declined toward later time points. In contrast, most other *IbCHR* genes exhibited a pronounced induction at 6 h, followed by a decrease at 12–24 h; several genes remained above the basal level at 48 h, indicating sustained ABA responsiveness.

Following MeJA treatment, *IbCHR* genes displayed distinct temporal patterns. For example, *IbCHR2* and *IbCHR3* increased strongly at 12 h and remained elevated thereafter, whereas *IbCHR6* showed a transient increase at 3 h. In addition, several genes (including *IbCHR4*, *IbCHR8*, *IbCHR10–IbCHR12*) showed higher expression at later time points, with *IbCHR10* exhibiting a gradual increase during 3–24 h and reaching its highest level at 48 h. Collectively, these results indicate that *IbCHR* genes respond to both ABA and MeJA treatments with gene-specific kinetics, supporting their potential involvement in ABA and JA regulatory pathways in sweet potato.

### 3.7 Subcellular localization of IbCHR10

**Figure 8:**
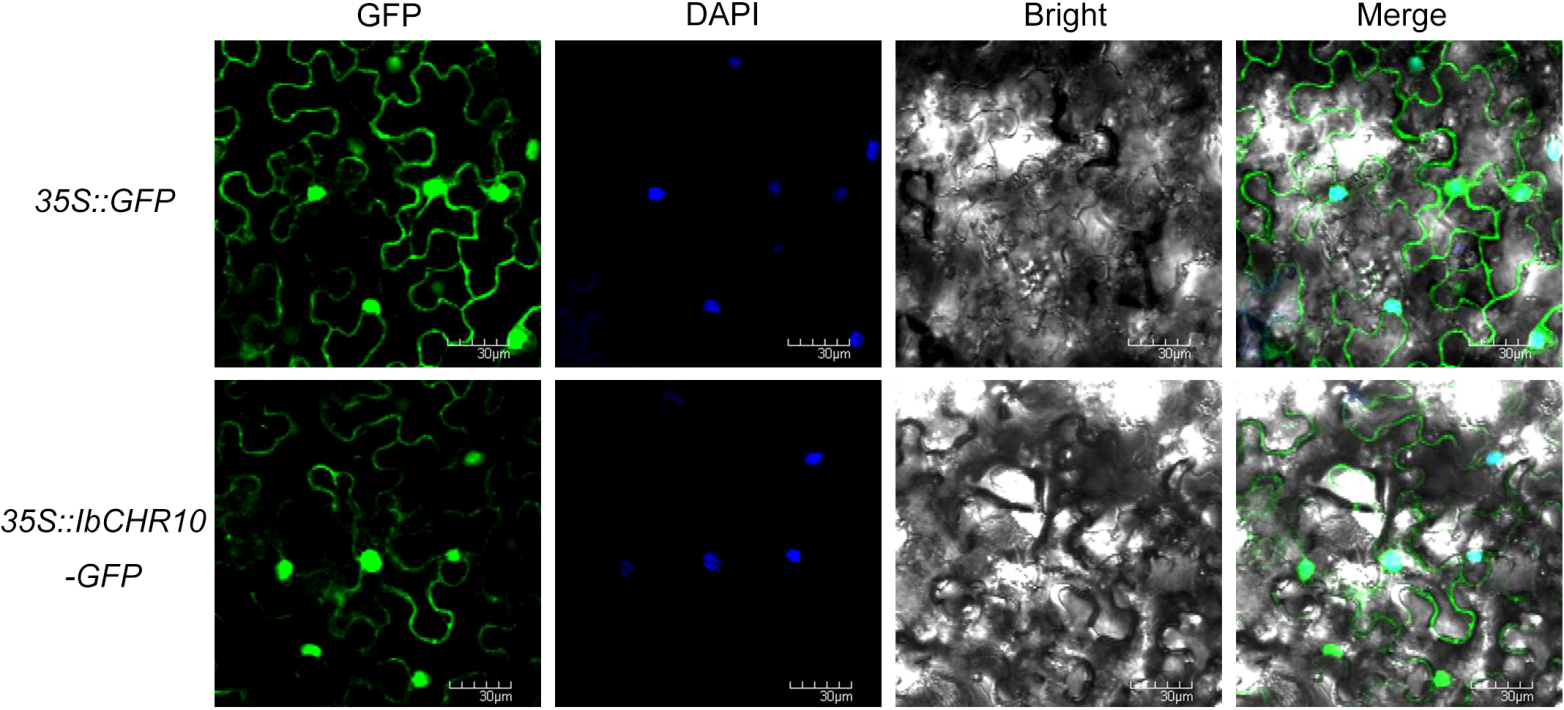
Subcellular localization of IbCHR10. 35S::IbCHR10-GFP and 35S::GFP were transiently expressed in *N. benthamiana* leaves. GFP fluorescence (green), DAPI-stained nuclei (blue), and bright-field images were captured by confocal microscopy. Merged images indicate that IbCHR10-GFP signals are present in the nucleus and cytoplasm. Bars, 30 μm.

IbCHR10 was selected for localization analysis because it exhibited rapid and sustained induction under salt stress in both RNA-seq screening and subsequent qRT-PCR validation, and was therefore prioritized for functional characterization in this study. To determine subcellular localization, IbCHR10 was fused to GFP under the CaMV 35S promoter (35S::IbCHR10-GFP) and transiently expressed in *N. benthamiana* epidermal cells via *Agrobacterium*-mediated infiltration. Confocal microscopy showed that the IbCHR10-GFP signal was detectable in both the nucleus (co-localizing with DAPI staining) and the cytoplasm. The overall distribution pattern of IbCHR10-GFP was very similar to that of free GFP, which was also broadly present in the nucleus and cytoplasm under the same experimental conditions (**Figure 8**). Therefore, while IbCHR10 is compatible with nuclear and cytoplasmic localization in this transient assay, the current results do not allow us to unambiguously distinguish specific targeting from diffuse distribution that may arise from strong CaMV 35S-driven expression.

Although C-terminal GFP fusion may theoretically interfere with C-terminal targeting signals or regulatory motifs, the absence of predicted C-terminal organellar targeting sequences in IbCHR10 reduces the likelihood that the fusion strategy altered its intrinsic localization. Nevertheless, because fusion orientation and expression level can influence subcellular distribution, complementary approaches will be required to further validate the subcellular localization of IbCHR10 in future studies.

### 3.8 Generation of *IbCHR10* overexpression transgenic sweet potato plants

**Figure 9:**
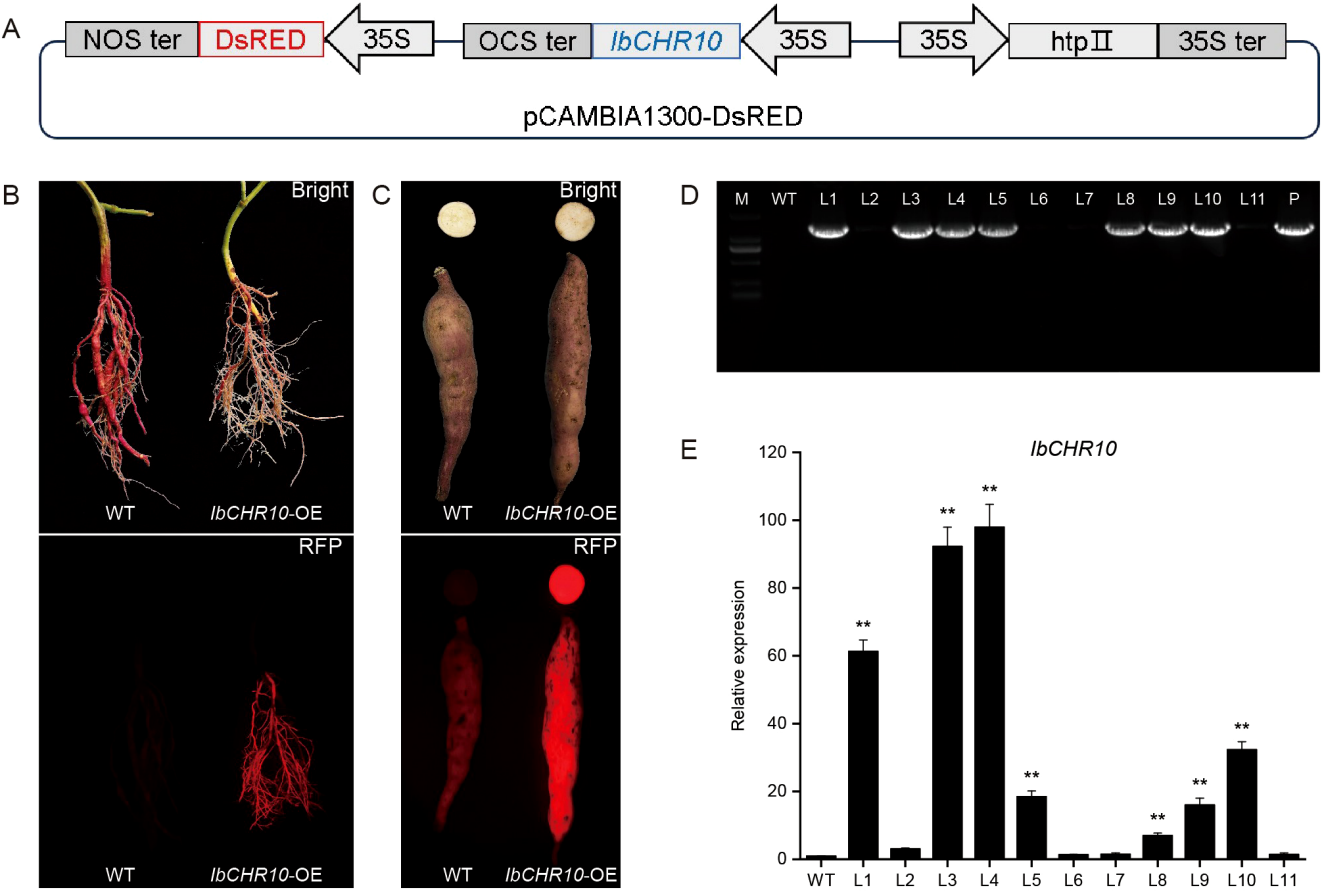
Generation and verification of *IbCHR10*-overexpressing transgenic sweet potato. **(A)** Schematic representation of the modified plant overexpression vector pCAMBIA1300-DsRed harboring the full-length *IbCHR10* coding sequence. **(B)** Fibrous roots with strong red fluorescence observed at the injection sites 6 weeks after *Agrobacterium rhizogenes* K599-mediated transformation of sweet potato cultivar ‘JS33’. **(C)** Transgenic sweet potato plants producing storage roots exhibiting red fluorescence signals, indicating successful genomic integration and expression of *IbCHR10*. **(D)** PCR analysis of the transgenic plants. Lane M: DL2000 DNA marker; Lane WT: ‘JS33’ plant as a negative control; Lane 1-11: possible *IbCHR10*-OE transgenic lines; Lane P: plasmid pCAMBIA1300-IbCHR10-DsRed as a positive control. **(E)** Relative expression levels of *IbCHR10* in transgenic lines determined by qRT-PCR analysis. Data are shown as mean ± SD of three independent biological replicates. ** indicates a significant difference compared with WT at P < 0.01.

The full-length *IbCHR10* CDS amplified from the salt-tolerant cultivar ‘JS26’ was cloned into the pCAMBIA1300-DsRed overexpression vector (**Figure 9A**) and introduced into *Agrobacterium rhizogenes* K599 for transformation of sweet potato cultivar ‘JS33’. In this vector, *IbCHR10* is driven by the CaMV 35S promoter, whereas DsRed is expressed from an independent 35S promoter as a fluorescent reporter. At approximately 6 weeks after injection, adventitious roots exhibiting strong red fluorescence were observed at the inoculation sites (**Figure 9B**). During subsequent growth, several transformed plants developed storage roots showing clear red fluorescence (**Figure 9C**), consistent with reporter expression in transformed tissues. In total, 20 plants were injected, and 7 independent PCR-positive lines (7/20) were identified (L1, L3, L4, L5, L8, L9, and L10) using WT plants as negative controls (**Figure 9D**), supporting genomic integration of the *IbCHR10* transgene. qRT-PCR further showed that *IbCHR10* transcript levels were elevated in these PCR-positive lines (**Figure 9E**). Based on transcript abundance, three lines (L1, L3, and L4) were selected for downstream phenotypic and physiological analyses under salt stress.

### 3.9 Functional characterization of *IbCHR10* under salt stress

**Figure 10:**
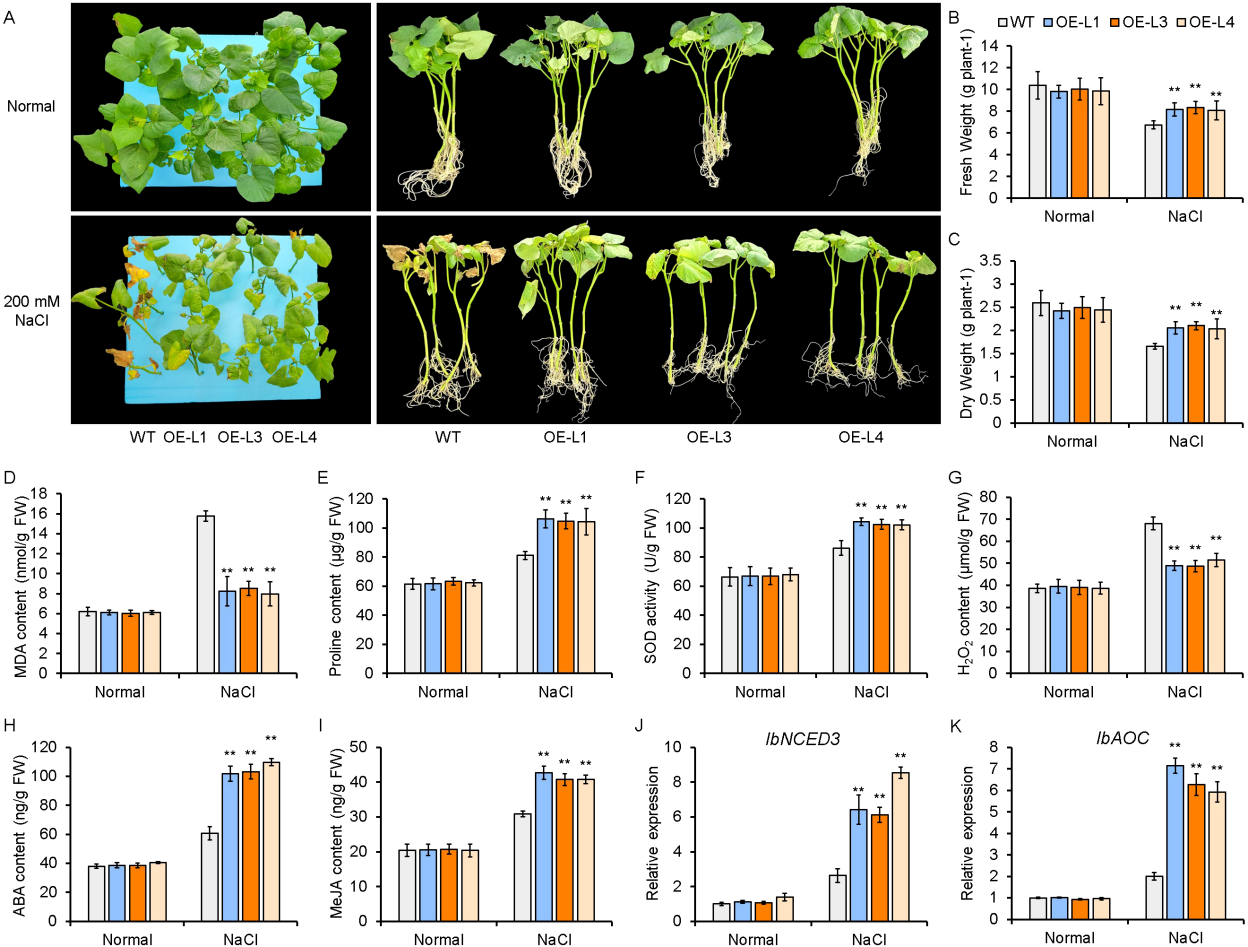
*IbCHR10* overexpression enhances salt tolerance in sweet potato. **(A)** Phenotypic performance of three independent *IbCHR10*-OE transgenic lines (L1, L3, and L4) and WT plants under 200 mM NaCl hydroponic conditions. **(B, C)** Fresh weight and dry weight of WT and *IbCHR10*-OE lines after salt treatment. **(D-G)** MDA and proline content, SOD activity, and H_2_O_2_ content of WT and *IbCHR10*-OE lines after salt treatment. **(H, I)** Endogenous ABA and MeJA contents in WT and *IbCHR10*-OE lines after salt treatment. **(J, K)** Relative expression levels of *IbNCED3* and *IbAOC* after salt treatment. Data are presented as mean ± SD (n = 3 biological replicates). Statistical significance was determined by two-way ANOVA (genotype × treatment) followed by Tukey’s multiple comparison test. **P < 0.01.

To evaluate the function of IbCHR10 in salt tolerance, three OE lines (L1, L3, and L4) and WT plants were assessed under NaCl stress using *in vitro* culture, pot, and hydroponic systems. Under 150 mM NaCl *in vitro*, OE lines showed stronger root regeneration than WT (**Supplementary Figure 3**). In pot assays with 200 mM NaCl irrigation, OE lines maintained higher fresh weight and longer roots than WT (**Supplementary Figure 4**). Consistently, under hydroponic treatment with 200 mM NaCl, all three OE lines outperformed WT, showing increased fresh weight and dry weight (**Figures 10A–C**).

To explore physiological changes associated with enhanced tolerance, several biochemical parameters were measured. Under normal conditions, no clear differences in ROS-related traits or antioxidant enzyme activity were observed between WT and OE lines. After salt treatment, OE lines exhibited reduced MDA content (**Figure 10D**), increased proline accumulation (**Figure 10E**) and SOD activity (**Figure 10F**), and decreased H_2_O_2_ levels (**Figure 10G**), consistent with improved osmotic adjustment and antioxidant capacity. We further examined stress hormone responses. Although ABA and MeJA levels were comparable between WT and OE plants under control conditions, salt treatment led to higher accumulation of both hormones in OE lines than in WT (**Figures 10H, I**). Correspondingly, *IbNCED3* (ABA biosynthesis) and *IbAOC* (JA biosynthesis) were upregulated in OE lines following salt treatment (**Figures 10J, K**). Collectively, these results suggest that IbCHR10 overexpression enhances salt tolerance in sweet potato, potentially through coordinated regulation of antioxidant defenses and ABA/JA-associated responses.

## 4 Discussion

DC1 domain proteins represent a unique class of cysteine/histidine-rich zinc-binding proteins (Hwang et al., 2014). These proteins are characterized by a conserved DC1domain that is structurally related to the C1 domains of animal protein kinase C (PKC), which mediate lipid-dependent signaling (Das and Rahman, 2014). Although C1 domains have been extensively characterized in animal systems (Das and Rahman, 2014), plant DC1 domain proteins remain less well studied. Nevertheless, accumulating evidence indicates that DC1 domain proteins participate in diverse processes, including signal transduction, development, and stress responses (D’Ippolito et al., 2017; Li et al., 2025; Hwang et al., 2014). In this study, we performed the first comprehensive genome-wide identification of the DC1 domain (CHR) protein gene family in cultivated sweet potato and its two diploid relatives (*I. trifida* and *I. triloba*). The similar number of CHR genes among these species suggests that this family is broadly conserved within *Ipomoea*, despite genome polyploidization in cultivated sweet potato. By contrast, the reported *CHR* gene numbers in Arabidopsis and tomato differ substantially (Bhaskar et al., 2015; Li et al., 2023), indicating that lineage-specific expansion or contraction of this family has occurred during plant evolution.

Synteny and duplication analyses further revealed that most *IbCHR* genes retained orthologous counterparts in both diploid relatives, suggesting that these loci have been subject to evolutionary constraint. Such patterns are consistent with the general role of segmental duplication in shaping plant gene families (Shukla et al., 2025). However, a subset of *IbCHR* genes lacked detectable collinearity with diploid genomes, which may reflect genome rearrangements or duplication-loss events associated with polyploidization. Structural analysis showed that most *CHR* genes are intron-poor, a feature commonly observed among stress-responsive genes and often associated with rapid transcriptional activation (Liu et al., 2021). In addition, promoter analysis revealed abundant cis-acting elements related to light responsiveness, phytohormone regulation, and abiotic stress. In particular, the widespread occurrence of ABA-responsive and JA-responsive motifs provides a plausible transcriptional basis for the hormone-dependent expression patterns observed for many *CHR* genes. Given that ABA and JA play central roles in plant responses to salinity, drought, and pathogen attack (Hong et al., 2013; Dar et al., 2015), *CHR* genes may participate in hormone-associated regulatory pathways that contribute to stress adaptation in sweet potato.

Expression profiling further supported functional diversification of CHR genes across tissues and stress conditions. Several *CHR* genes displayed preferential expression in fibrous roots, which are primary sites for sensing soil conditions and stress cues (Ghosh and Xu, 2014), consistent with their potential involvement in root-associated environmental responses. By contrast, many *IbCHR* genes exhibited relatively low expression in storage roots, which may reflect tissue-specific regulatory programs; however, their roles in storage-root development remain to be determined. In addition, because tissue-expression profiles of *I. trifida* and *I. triloba* were derived from public RNA-seq datasets, whereas *I. batatas* expression was assessed by qRT-PCR in this study, these datasets are not directly quantitatively comparable. Therefore, our cross-species interpretation is focused on qualitative expression trends rather than absolute expression levels.

Salt stress exerts a two-phase detrimental effect on plants, consisting of an early osmotic stress phase that restricts water uptake and growth, followed by ionic stress caused by Na^+^ and Cl^-^ accumulation that leads to oxidative damage (Polash et al., 2019; Hao et al., 2021). Comparative expression analysis showed that *IbCHR10* was progressively induced within 0–12 h after salt treatment, with a rapid and strong early induction in the salt-tolerant cultivar ‘JS26’ but only weak induction in the salt-sensitive cultivar ‘NL54’. This genotype-dependent early responsiveness is consistent with a role for *IbCHR10* in salt tolerance, potentially related to early osmotic-stress signaling. In contrast, *IbCHR7* exhibited opposite expression trends between the two cultivars, suggesting that different CHR family members may play divergent roles in salt stress responses. Such functional divergence within gene families has also been reported in sweet potato, for example among bHLH transcription factors, overexpression of *IbbHLH66* enhances drought tolerance in transgenic sweet potato, whereas overexpression of *IbbHLH118* reduces drought tolerance (Xue et al., 2022).

Functional analyses provided direct evidence that *IbCHR10* contributes to salt tolerance. Overexpression of *IbCHR10* enhanced plant performance under salt stress *in vitro*, in pots, and in hydroponic culture, demonstrating its effect across different growth systems. At the physiological level, *IbCHR10*-OE lines showed reduced accumulation of MDA and H_2_O_2_ together with increased SOD activity, indicating enhanced antioxidant capacity and alleviated oxidative damage (Kesawat et al., 2023). In parallel, elevated proline levels in OE plants suggest improved osmotic adjustment, further supporting cellular homeostasis under salinity. Moreover, *IbCHR10* overexpression led to increased accumulation of ABA and JA under salt stress, accompanied by higher transcript levels of the biosynthetic genes *IbNCED3* and *IbAOC*. These coordinated changes are consistent with enhanced activation of hormone-mediated stress response pathways, which play pivotal roles in plant adaptation to salinity and drought (Hong et al., 2013; Dar et al., 2015).

Several limitations of the present study should be noted. In this study, functional validation was performed by expressing the JS26-derived *IbCHR10* CDS in the JS33 background under a constitutive promoter. Therefore, the assay primarily evaluated the functional effect of the coding sequence, while cultivar-specific differences in the native *IbCHR10* promoter or other cis-regulatory elements were not directly tested. In addition, trans-acting regulatory differences in the JS33 background may affect the magnitude of downstream responses. Future comparisons of *IbCHR10* alleles and native promoter regions between JS26 and JS33 will help further clarify the contribution of sequence polymorphisms and cultivar-specific regulatory contexts. We also acknowledge that an empty-vector and/or DsRed-only transgenic control was not included in the present study, which limits our ability to fully exclude potential transformation- or marker-related effects. Although multiple independent *IbCHR10*-overexpressing lines showed consistent phenotypic and physiological responses and were confirmed by PCR and qRT-PCR, future experiments including appropriate transgenic controls will be necessary to strengthen causal inference. In addition, although transgene presence and *IbCHR10* expression were verified in multiple tissues and developmental stages, a continuous quantitative time-course analysis across all tissues was not performed in this study and will be addressed in future work.

Collectively, our results identify IbCHR10 as a promising regulator associated with multiple stress-responsive processes, including redox homeostasis, osmotic balance, and hormone-associated signaling. Although the direct molecular mechanism remains to be elucidated, the coordinated physiological, transcriptional, and hormonal responses observed in *IbCHR10*-overexpressing plants strongly support its contribution to salt tolerance in sweet potato. These findings provide both a functional candidate gene and a mechanistic framework that may facilitate future efforts to improve salt resilience in sweet potato through molecular breeding and biotechnological approaches.

## 5 Conclusions

In this study, we conducted the first comprehensive genome-wide characterization of the DC1 domain (*CHR*) gene family in sweet potato (*I. batatas*) and its diploid relatives (*I. trifida* and *I. triloba*). Comparative genomic and expression analyses revealed tissue-preferential and stress-responsive transcriptional patterns, indicating functional diversification of *CHR* genes during development and environmental adaptation. Among the identified members, *IbCHR10* was strongly responsive to salt stress. Functional validation demonstrated that overexpression of *IbCHR10* enhances salt tolerance in sweet potato, supporting its positive regulatory role in abiotic stress responses. Subcellular localization analysis indicated that IbCHR10 showed a nucleus/cytoplasm localization pattern under transient expression conditions, providing a basis for further investigation of its cellular role in salt-stress responses. Collectively, our findings provide a systematic framework for understanding DC1 domain proteins in sweet potato and identify IbCHR10 as a promising candidate for improving salt tolerance through molecular breeding strategies.

## Data Availability

The datasets presented in this study can be found in online repositories. The names of the repository/repositories and accession number(s) can be found in the article/**Supplementary Material**.
