# Supplementary material for: Genome-wide analysis of DC1 domain proteins in Ipomoea species reveals IbCHR10 as a positive regulator of salt tolerance in sweet potato
Source: Front Plant Sci. 2026 Mar 16;17:1780326. doi: 10.3389/fpls.2026.1780326 (PMC13033517; doi:10.3389/fpls.2026.1780326)
Supplement: Supplementary file 1 [file DataSheet1.docx]

**
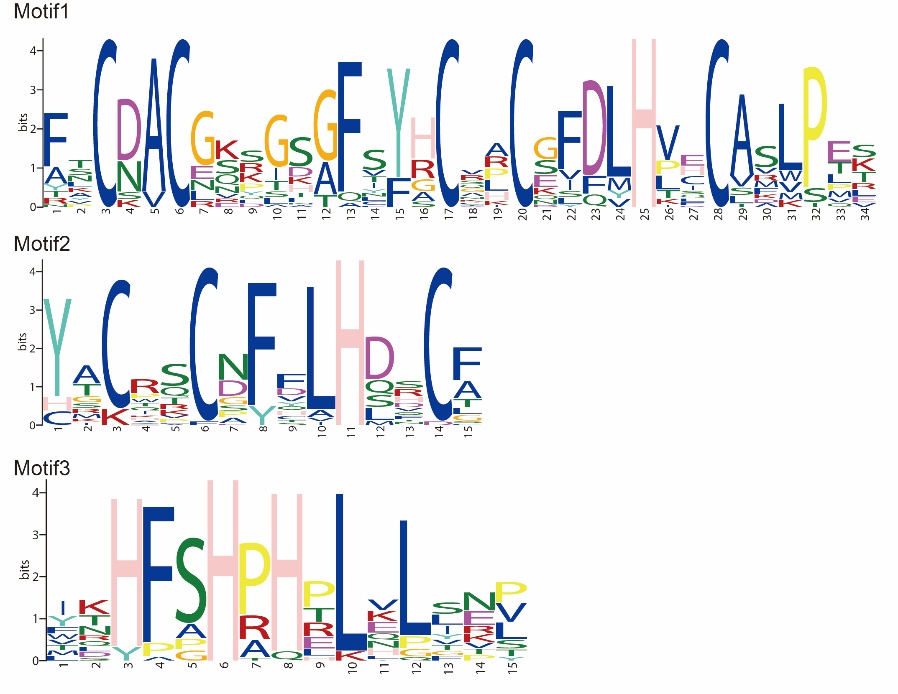
**

**Fig. S1 Conservation analysis of amino acid residues in the core DC1 domain of CHR proteins**

Conserved residues within the DC1 domain were identified by multiple sequence alignment and visualized to highlight highly conserved amino acids among *CHR* gene family members


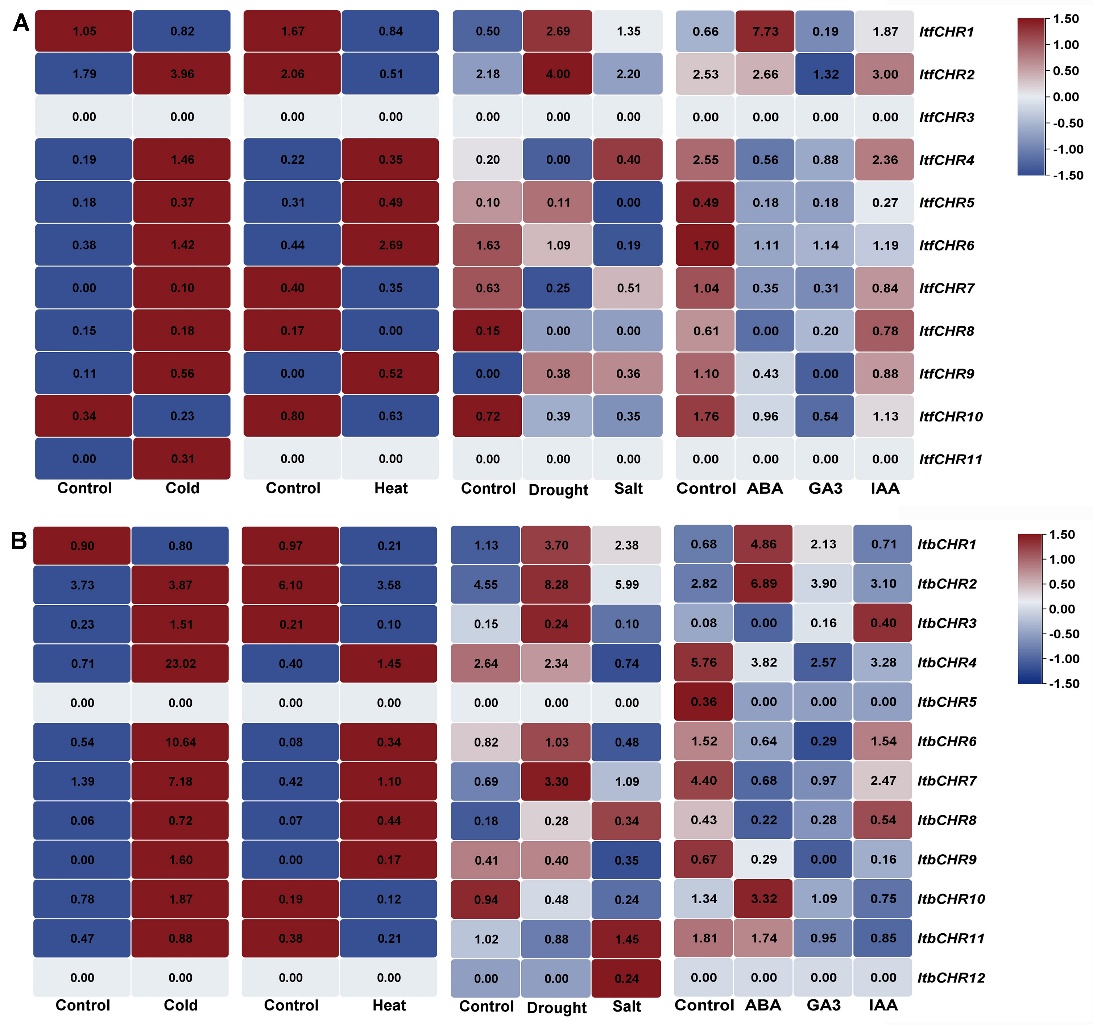


**Fig. S2 Expression profiles of *CHR* genes in *I. trifida* and *I. triloba* under abiotic stresses and hormone treatments**

(A) Heatmap showing expression patterns of *ItfCHR* genes in response to cold, heat, drought, salt, ABA, GA₃, and IAA treatments. (B) Heatmap showing expression patterns of *ItbCHR* genes under the same conditions.

**
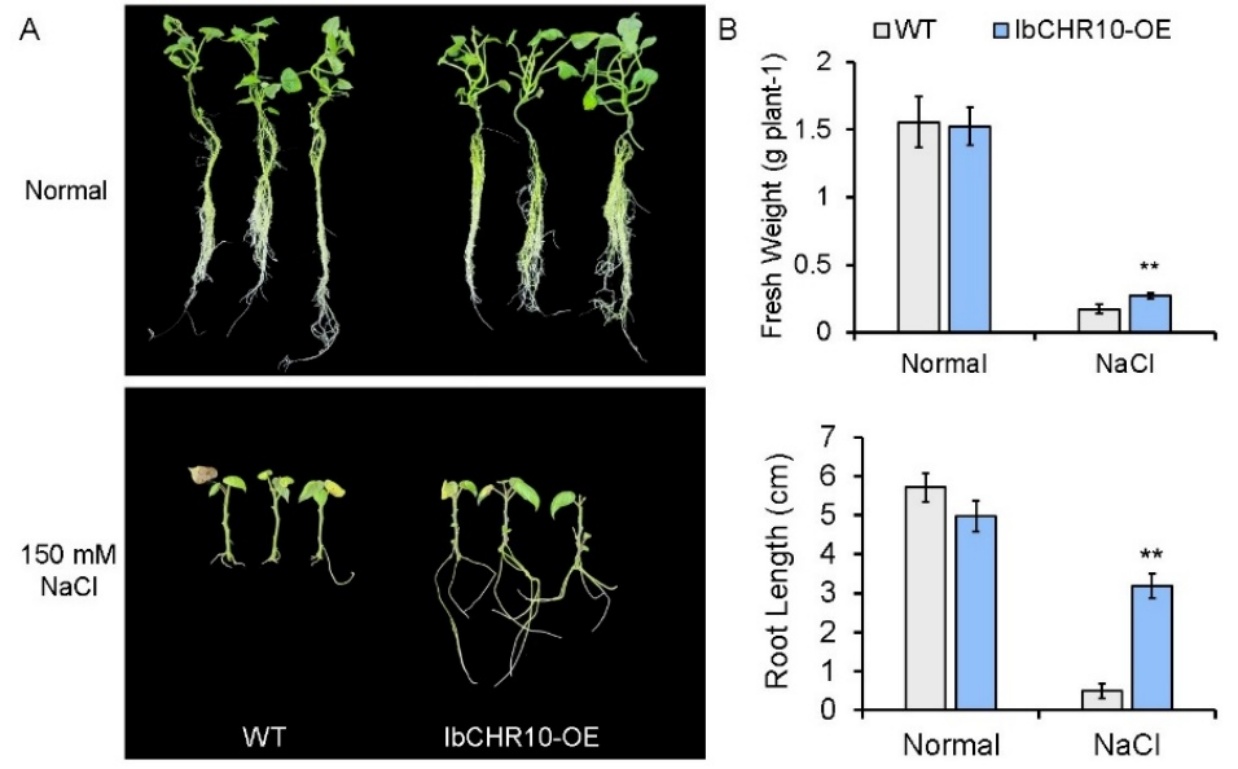
**

**Fig. S3 Salt tolerance of *IbCHR10*-overexpressing (OE) sweetpotato plants in vitro**

(A) Phenotypic comparison of WT and *IbCHR10*-OE plants under normal and salt stress (150 mM NaCl) conditions. (B) Quantitative analysis of fresh weight and root length in WT and *IbCHR10*-OE plants under normal and NaCl treatment conditions. Data are presented as mean ± SD (*n* = 3 biological replicates). Statistical significance was determined by two-way ANOVA (genotype × treatment) followed by Tukey’s multiple comparison test. **, P < 0.01.


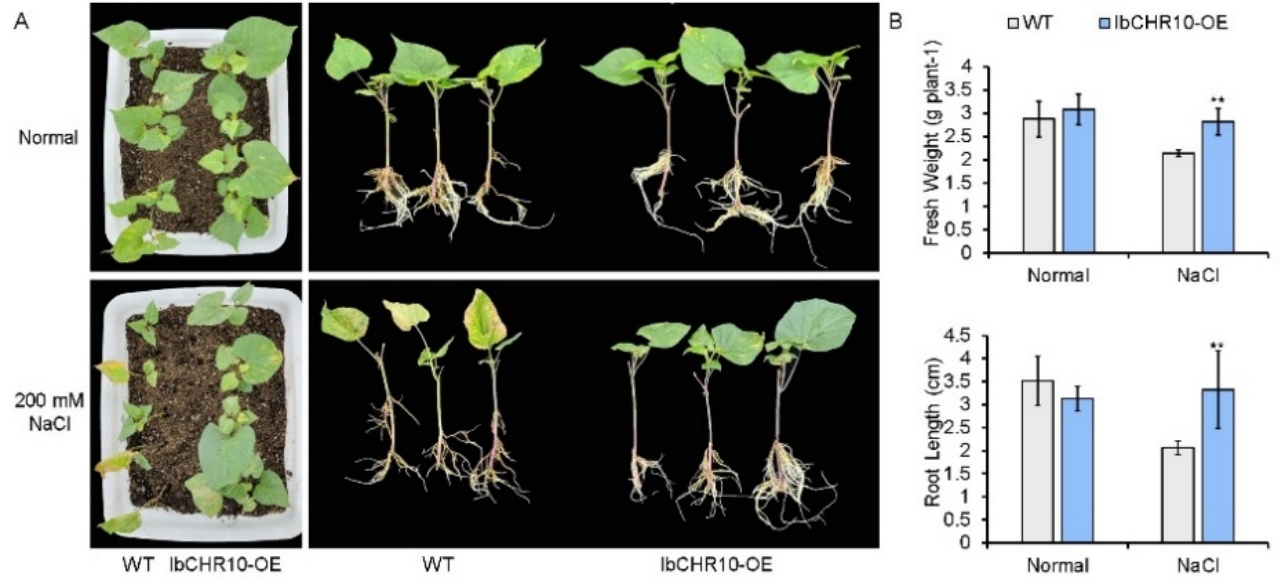


**Fig. S4 Salt tolerance of *IbCHR10*-OE sweetpotato plants in pot experiments**

(A) Phenotypic comparison of WT and *IbCHR10*-OE plants under normal and salt stress (200 mM NaCl) conditions. (B) Quantitative analysis of fresh weight and root length in WT and *IbCHR10*-OE plants under normal and NaCl treatment conditions. Data are presented as mean ± SD (*n* = 3 biological replicates). Statistical analysis was performed as described in Fig. S3.

**Table S1 Physicochemical properties and predicted subcellular localization of CHR proteins in sweetpotato (*I. batatas*) and its diploid relatives (*I. trifida* and *I. triloba*)**

| **Gene name** | **Protein size (aa)** | **MW (kDa)** | **pI** | **Instability index** | | **aliphatic index** | **GRAVY** | **Subcellular location** |
| --- | --- | --- | --- | --- | --- | --- | --- | --- |
| IbCHR1 | 276 | 29.39128 | 8.19 | | 58.2 | 76.38 | -0.252 | nucleus |
| IbCHR2 | 265 | 28.92569 | 6.72 | | 50.41 | 67.81 | -0.231 | nucleus |
| IbCHR3 | 395 | 40.20312 | 8.86 | | 57.51 | 74.15 | -0.165 | chloroplast |
| IbCHR4 | 202 | 22.60467 | 8.99 | | 71.94 | 74.46 | -0.666 | nucleus |
| IbCHR5 | 401 | 44.28687 | 6.02 | | 53.78 | 58.63 | -0.424 | peroxisome |
| IbCHR6 | 167 | 18.57345 | 8.63 | | 62.85 | 84.85 | -0.275 | mitochondria |
| IbCHR7 | 239 | 27.48434 | 7.87 | | 46.46 | 65.69 | -0.489 | peroxisome |
| IbCHR8 | 168 | 18.9265 | 11.44 | | 75.14 | 49.52 | -0.732 | nucleus |
| IbCHR9 | 127 | 14.111 | 10.31 | | 71.54 | 52.28 | -0.618 | nucleus |
| IbCHR10 | 349 | 39.0012 | 6.48 | | 59.83 | 68.22 | -0.352 | nucleus |
| IbCHR11 | 342 | 35.95999 | 9.42 | | 48.14 | 68.57 | -0.3 | mitochondria |
| IbCHR12 | 201 | 22.73858 | 6.26 | | 61.15 | 55.82 | -0.534 | mitochondria |
| ItfCHR1 | 350 | 36.70682 | 9.37 | | 49.42 | 68.69 | -0.28 | nucleus |
| ItfCHR2 | 242 | 27.47217 | 9.16 | | 42.18 | 69.34 | -0.25 | cytoplasm |
| ItfCHR3 | 516 | 56.87765 | 6.42 | | 51.28 | 63.6 | -0.275 | nucleus |
| ItfCHR4 | 241 | 26.72323 | 5.87 | | 45.44 | 61.2 | -0.315 | nucleus |
| ItfCHR5 | 403 | 44.64433 | 5.97 | | 52.62 | 58.83 | -0.453 | chloroplast |
| ItfCHR6 | 235 | 26.04136 | 5.93 | | 44.37 | 66.94 | -0.332 | mitochondria |
| ItfCHR7 | 423 | 43.97519 | 7.22 | | 59.69 | 70.09 | -0.234 | nucleus |
| ItfCHR8 | 280 | 30.11999 | 6.81 | | 48.87 | 66.96 | -0.237 | nucleus |
| ItfCHR9 | 321 | 35.15709 | 8.12 | | 47.26 | 75.39 | -0.269 | nucleus |
| ItfCHR10 | 233 | 26.25759 | 6.42 | | 67.06 | 67.85 | -0.473 | nucleus |
| ItfCHR11 | 196 | 22.15399 | 8.47 | | 52.1 | 63.32 | -0.564 | nucleus |
| ItbCHR1 | 350 | 36.77991 | 9.36 | | 46.73 | 68.94 | -0.288 | nucleus |
| ItbCHR2 | 244 | 27.61626 | 9.15 | | 40.95 | 67.58 | -0.293 | cytoplasm |
| ItbCHR3 | 385 | 43.07896 | 6.94 | | 55.55 | 65.14 | -0.409 | nucleus |
| ItbCHR4 | 241 | 26.69823 | 5.92 | | 47.93 | 62.45 | -0.291 | nucleus |
| ItbCHR5 | 160 | 17.94932 | 7.2 | | 47.42 | 67.81 | -0.426 | mitochondria |
| ItbCHR6 | 364 | 40.06436 | 6.56 | | 60.35 | 59.26 | -0.43 | chloroplast |
| ItbCHR7 | 235 | 26.02823 | 5.69 | | 45.23 | 66.09 | -0.331 | cytoplasm |
| ItbCHR8 | 419 | 43.47578 | 8.16 | | 58.74 | 70.53 | -0.21 | nucleus |
| ItbCHR9 | 266 | 28.75362 | 7 | | 48.57 | 70.15 | -0.142 | nucleus |
| ItbCHR10 | 323 | 35.49846 | 8.29 | | 48.37 | 72.2 | -0.364 | nucleus |
| ItbCHR11 | 196 | 22.44848 | 6.09 | | 59.72 | 50.31 | -0.492 | nucleus |
| ItbCHR12 | 202 | 22.63656 | 5.7 | | 48.83 | 56.93 | -0.61 | chloroplast |

**Table S2 Primer sequences**

| **Primer name** | **Sequence** |
| --- | --- |
| IbActin-F | CTGGTGTTATGGTTGGGATGG |
| IbActin-R | GGGGTGCCTCGGTAAGAAG |
| IbCHR1-qRT-F | GTGCAGGTACAGGGGCAG |
| IbCHR1-qRT-R | CCTCCCCATTGCGGTGTT |
| IbCHR2-qRT-F | TCGACACAGCCCACGTTC |
| IbCHR2-qRT-R | TCGTTTCCCACAGGCGTC |
| IbCHR3-qRT-F | TTATTGAACGCCGCCGGT |
| IbCHR3-qRT-R | ACCACCTCCCATCAACGC |
| IbCHR4-qRT-F | ACGTTCACCTCCACTGCG |
| IbCHR4-qRT-R | CCGCCATCCTCATCACCC |
| IbCHR5-qRT-F | GCCTGTCTGCAGAAGGGG |
| IbCHR5-qRT-R | CTGCACTTTCTCCGGCCA |
| IbCHR6-qRT-F | AAATCCCACCCCAGCACG |
| IbCHR6-qRT-R | CAGACGCGGCAGGAGTAG |
| IbCHR7-qRT-F | CCGAACGTGAGAGGGAGC |
| IbCHR7-qRT-R | AACCCAGCCGTCTCTCCT |
| IbCHR8-qRT-F | CACGTGCACCAAGCCAAG |
| IbCHR8-qRT-R | CGCACGTGAACTCCCGAT |
| IbCHR9-qRT-F | CGCAACTCAACGGCAAGC |
| IbCHR9-qRT-R | CCACCACCACCTCTGCAA |
| IbCHR10-qRT-F | ACATGTGCCAAAAATGCC |
| IbCHR10-qRT-R | TCTGGGACGAGAGGTCTA |
| IbCHR11-qRT-F | TGCAAAGAGTACGGCGCA |
| IbCHR11-qRT-R | GGGAGGAGAGAGGGCACA |
| IbCHR12-qRT-F | CACGTGCACCAAGCCAAG |
| IbCHR12-qRT-R | CGCACGTGAACTCCCGAT |
| IbNCED3-qRT-F | AGAAGCAGGGCAAATAAACAAG |
| IbNCED3-qRT-R | CCGTCGCCGTACCTAAACTC |
| IbAOC-qRT-F | TAAGCTCCCCTCCACCTACC |
| IbAOC-qRT-R | GTAACCGATCTTTTGGCGGC |
| IbCHR10-CL-F | ATGGAGTATAAGCATTTCAGCC |
| IbCHR10-CL-R | TCACTCATGAAATCGAGTGAAG |
